# Isoniazid-Derived Hydrazones Featuring Piperazine/Piperidine Rings: Design, Synthesis, and Investigation of Antitubercular Activity

**DOI:** 10.3390/biom15091305

**Published:** 2025-09-11

**Authors:** Esma Özcan, Siva Krishna Vagolu, Rasoul Tamhaev, Christian Lherbet, Lionel Mourey, Tone Tønjum, Miyase Gözde Gündüz, Şengül Dilem Doğan

**Affiliations:** 1 Department of Basic Sciences, Faculty of Pharmacy, Erciyes University, 38039 Kayseri, Turkey; esmaozcan038@gmail.com; 2 Unit for Genome Dynamics, Department of Microbiology, University of Oslo, 0316 Oslo, Norway; tone.tonjum@medisin.uio.no; 3 Research Institute of Internal Medicine, Oslo University Hospital, 0424 Oslo, Norway; 4 Laboratoire de Synthèse et Physico-Chimie de Molécules d’Interêt Biologique, LSPCMIB, Université de Toulouse, CNRS, 118 Route de Narbonne, 31062 Toulouse, France; rasoul.tamhaev@gmail.com (R.T.); christian.lherbet@univ-tlse3.fr (C.L.); 5 Institut de Pharmacologie et de Biologie Structurale, Université de Toulouse, CNRS, 205 route de Narbonne, BP 64182, 31077 Toulouse, Cedex 4, France; lionel.mourey@ipbs.fr; 6 Unit for Genome Dynamics, Department of Microbiology, Oslo University Hospital, 0424 Oslo, Norway; 7 Department of Pharmaceutical Chemistry, Faculty of Pharmacy, Hacettepe University, Sıhhiye, 06100 Ankara, Turkey; miyasegunduz@yahoo.com

**Keywords:** antimycobacterial, InhA, molecular modeling, *Mycobacterium tuberculosis*, cytotoxicity, *N*-acylhydrazone

## Abstract

Isoniazid (isonicotinic acid hydrazide, INH) is a key drug used to treat tuberculosis (TB), which continues to be the world’s most lethal infectious disease. Nevertheless, the efficacy of INH has diminished because of the emergence of *Mycobacterium tuberculosis* (*Mtb*) strains that are resistant to INH. Our goal in this study was to modify INH to reduce this significant resistance chemically. We synthesized INH-based hydrazones (**IP1**–**IP13**) through the reaction of INH with in-house obtained benzaldehydes carrying a piperidine or piperazine ring in refluxing ethanol. Upon confirmation of their proposed structures by various spectral techniques, **IP1**–**IP13** were evaluated for their antimycobacterial capacity against *Mtb* H37Rv strain and INH-resistant clinical isolates with *katG* and *inhA* mutations using the Microplate Alamar Blue Assay (MABA). The compounds were additionally tested for their cytotoxicity. The obtained data indicated that the compounds with moderately increased lipophilicity compared to INH (**IP7**–**IP13**) were promising antitubercular drug candidates, exhibiting drug-like properties and negligible cytotoxicity. Out of these, **IP11** (*N*′-(4-(4-cyclohexylpiperazin-1-yl)benzylidene)isonicotinohydrazide) emerged as the most promising derivative, demonstrating the lowest MIC values against all *Mtb* strains tested. Subsequently, the target molecules were evaluated for their capacity to inhibit enoyl acyl carrier protein reductase (InhA), the main target enzyme of INH. Except for **IP11** demonstrating 81% InhA inhibition at a concentration of 50 μM, direct InhA inhibition was shown not to be the primary mechanism responsible for the antitubercular activity of the compounds. The binding mechanism of **IP11** to InhA was analyzed through molecular docking and molecular dynamics simulations. Altogether, our research identified a novel approach to modify INH to address the challenges posed by the rising prevalence of drug-resistant *Mtb* strains.

## 1. Introduction

*Mycobacterium tuberculosis* (*Mtb*) is the main causative agent of tuberculosis (TB), a highly contagious disease that has impacted humanity for centuries [1]. According to the recent World Health Organization (WHO) Global TB Report, TB still ranks among the leading causes of death related to infectious agents [2]. The effectiveness of the treatment regimen, primarily consisting of four first-line drugs (isoniazid, rifampicin, pyrazinamide, and ethambutol), is often jeopardized by the emergence of drug-resistant strains of *Mtb* [3]. *Mtb* cells that are resistant to isoniazid and rifampicin are defined as being multidrug-resistant (MDR) and are the cause of MDR-TB, which is very difficult, if possible, to treat. To combat drug-resistant strains of *Mtb*, the primary strategies involve optimizing the use of current agents, developing new drug candidates, and repurposing existing marketed drugs [4].

Isoniazid (isonicotinic acid hydrazide, INH) remains the frontline medication for tuberculosis treatment today [5]. As a prodrug, once inside the *Mtb* cell, INH needs catalase-peroxidase (KatG) activation to inhibit its target enzyme, enoyl-acyl carrier protein reductase (InhA), essential for synthesizing mycolic acid [6,7]. Consequently, the clinical success of INH against drug-resistant TB is frequently hampered by mutations in *katG* and the *inhA* promoter in the *Mtb* genome [8].

**Figure 1:**
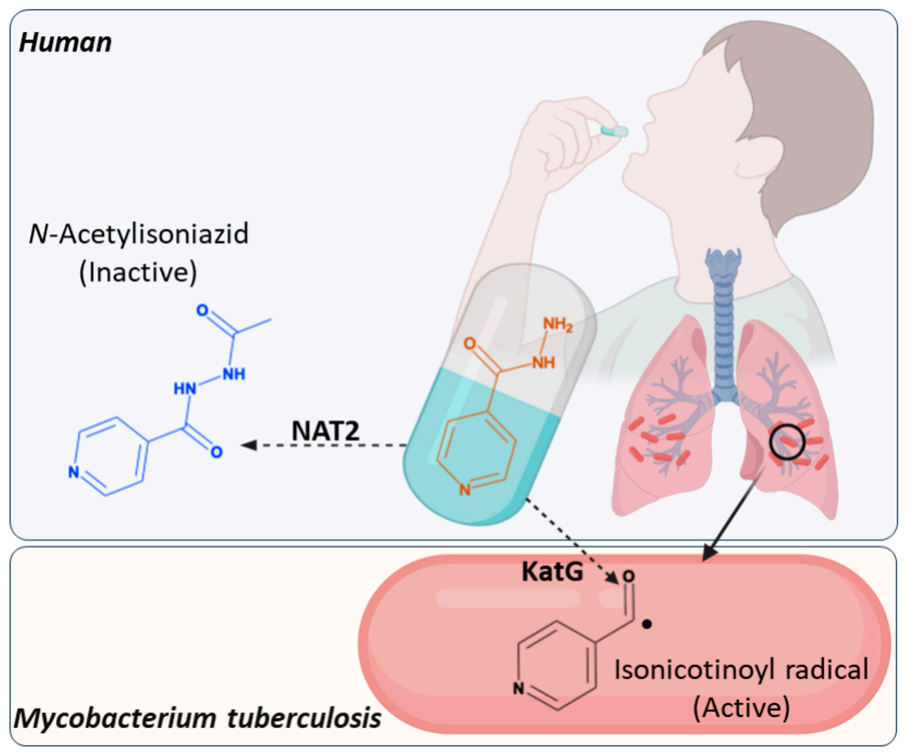
Illustrative representation of the mechanisms that result in the activation (inside *M. tuberculosis* cells) and deactivation of INH (inside human macrophages).

Inside macrophages of the exclusive human TB host, INH often undergoes metabolism through *N*-acetyltransferase 2 (NAT2), which deactivates INH by transferring an acetyl group from acetyl coenzyme A (acetyl-CoA) to the terminal nitrogen of its hydrazide functionality. Host genetic variations in the expressed NAT2 enzyme result in varying acetylation rates, which generate differences in efficient INH blood concentrations among individuals undergoing TB treatment [9]. The steps for activating and deactivating INH are outlined schematically in Figure 1.

INH as a cornerstone drug plays a significant role in the standardized TB treatment protocols endorsed by the WHO. Nevertheless, poor treatment adherence or incomplete therapy frequently results in the development of INH-resistant *Mtb* strains [10]. As a result, chemically altering INH is regarded as a rational approach for developing new antitubercular drugs to address the rising resistance. Particularly, modifications enabling INH to exert its antitubercular effect without requiring KatG activation or preventing the effect of the acetylation of the hydrazide functionality could open new pathways for antimycobacterial drug development [11].

**Figure 2:**
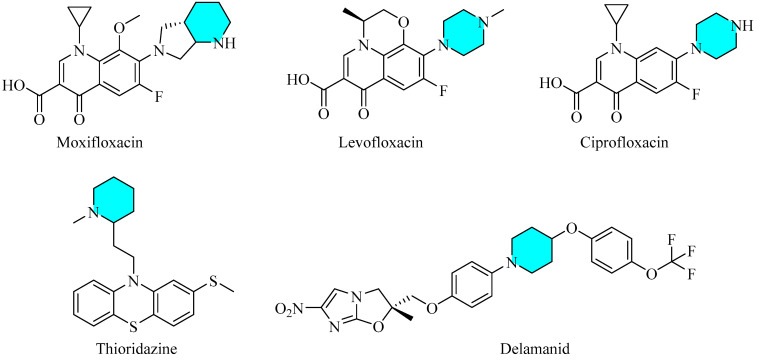
Chemical structures of drug molecules with piperazine or piperidine rings demonstrating activity against *Mycobacterium tuberculosis*.

Piperidine and piperazine are six-membered heterocycles featuring one and two nitrogen atoms, respectively. Due to their diverse biological properties, they serve as important structural components in drug design [12,13]. Notably, they are present in the chemical structures of drug molecules like fluoroquinolones [14], thioridazine [15], and delamanid [16], which are utilized for treating tuberculosis (Figure 2).

**Figure 3:**
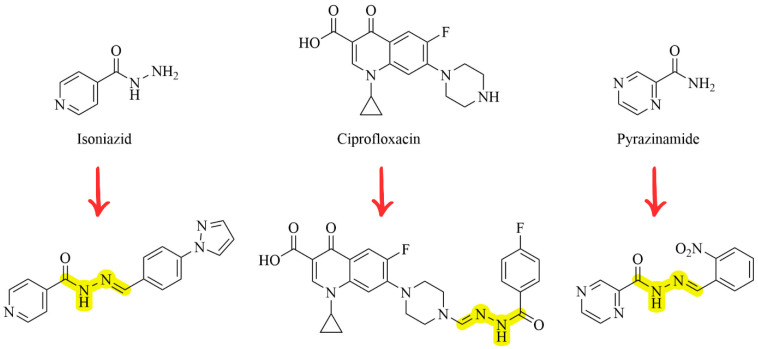
Hydrazone derivatives of some commercially available anti-TB drugs possessing antimycobacterial activity. Hydrazone moiety is highlighted in yellow.

Molecules with hydrazone functionality (-NHN=CH-) draw significant interest due to their frequently reported anti-infective properties [17]. Furthermore, its ability to inhibit mycobacterial pathogens makes hydrazone a significant structural element contributing to the development of new anti-TB agents [18]. Consequently, synthesizing hydrazone derivatives from commercial antitubercular agents such as isoniazid [19], ciprofloxacin [20], and pyrazinamide [21] appears to be an effective and non-toxic approach for identifying new desired drug candidates for TB treatment (Figure 3). Recently, synthesizing hydrazone derivatives of INH has remained a popular strategy for developing new antitubercular agents that are also effective against drug-resistant strains of *Mtb* [22,23,24,25].

**Figure 4:**
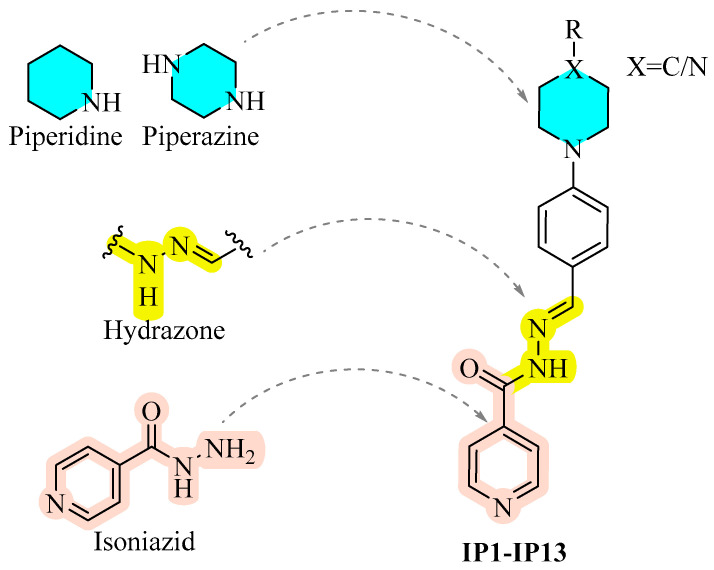
Design strategy for INH-based hydrazones carrying piperidine or piperazine rings (**IP1**–**IP13**).

Considering the abovementioned factors, we aimed to generate new antitubercular agents (**IP1**–**IP13**) by connecting the hydrazide group of INH to piperidine/piperazine rings via a benzylidene linker, resulting in the formation of hydrazone functionality (Figure 4). We then evaluated the antitubercular activity of **IP1**–**IP13** against the reference *Mtb* strain H37Rv and INH-resistant clinical isolates with *katG* and *inhA* promoter mutations.

## 2. Materials and Method

### 2.1. Chemistry

#### 2.1.1. Experimental

All chemicals and solvents were obtained from Sigma-Aldrich and Merck. Melting points were measured using a Schmelzpunktbestimmer 9300 SMP II apparatus (uncorrected). Syntheses were performed with Heidolph magnetic stirrers. Analytical TLC was carried out on Merck silica gel 60 F_254_ plates using a methanol: ethyl acetate mobile phase at 25 °C with UV detection at 254 nm. NMR (^1^H at 400 MHz, ^13^C at 100 MHz) spectra in DMSO-*d*_6_ were recorded on BRUKER AM 400 spectrometer at Erciyes University TAUM and HRMS data were obtained using Agilent 1200 Infinity Series HPLC system coupled with a 6530 Accurate-Mass Q-TOF LC/MS (Agilent Technologies, Santa Clara, CA, USA) at Atatürk University DAYTAM. Supporting Information included NMR and HRMS spectra.

##### Preparation of Benzaldehydes Carrying Piperazine/Piperidine Derivatives (**III**)

A solution of 4-fluorobenzaldehyde (**I**) (1 mmol) in 20 mL DMF was mixed with the piperazine or piperidine derivatives (**II**) (1 mmol) and potassium carbonate (1.2 mmol). The final mixture was heated under reflux in an oil bath for 18 h. Afterward, water was added into the reaction mixture, and the precipitate formed was collected by filtration. The resulting solid was utilized in the subsequent steps without any additional purification [26].

##### General Procedure for the Synthesis of **IP1**–**IP13**

A solution of piperidine/piperazine-carrying benzaldehyde (**III**) (1 mmol) in 10 mL ethanol was mixed with INH (**IV**) (1.1 mmol). The reaction mixture was heated under reflux in an oil bath until the starting materials were completely consumed. After cooling to room temperature, the resulting precipitate was filtered, washed with ethanol, and dried. The crude product was then purified by recrystallization from ethanol [27].

***N*′-(4-(4-Phenylpiperazin-1-yl)benzylidene)isonicotinohydrazide (IP1)**

Yellow solid, yield 50%, mp: 303–304 °C, Rf = 0.5 (MeOH/EtOAc 9:1). ^1^H NMR (400 MHz, DMSO-*d*_6_) *δ*_H_ 11.89 (s, 1H, -NH, D_2_O exchangeable), 8.78 (s, 2H, Ar-H), 8.35 (s, 1H, =CH), 7.83 (s, 2H, Ar-H), 7.62 (d, *J* = 8.1 Hz, 2H, Ar-H), 7.37–7.21 (m, 2H, Ar-H), 7.21–6.93 (m, 4H, Ar-H), 6.82 (d, *J* = 6.7 Hz, 1H, Ar-H), 3.29 (s, 4H, -C**H_2_**-N), (remaining piperazine signal overlapped with solvent). ^13^C NMR (100 MHz, DMSO-*d*_6_) *δ*_C_ 161.7 (C=O), 152.6, 151.2 (-C=N), 150.5, 149.9, 129.5, 129.1, 124.3, 122.1, 119.8, 116.2, 115.2, 113.8, 48.6 (piperazine-CH), 47.6 (piperazine-CH). HRMS-ESI (*m*/*z*): Calcd. for C_23_H_24_N_5_O (M+H)^+^: 386.1981; Found: 386.1980.

***N*′-(4-(4-(2-Fluorophenyl)piperazin-1-yl)benzylidene)isonicotinohydrazide (IP2)**

Yellow solid, yield 62%, mp: 296–297 °C, Rf = 0.75 (MeOH/EtOAc 8:2). ^1^H NMR (400 MHz, DMSO-*d*_6_) *δ*_H_ 11.85 (s, 1H, -NH, D_2_O exchangeable), 8.77 (d, *J* = 5.8 Hz, 2H, Ar-H), 8.36 (s, 1H, =CH), 8.02–7.75 (m, 2H, Ar-H), 7.62 (d, *J* = 8.7 Hz, 2H, Ar-H), 7.27–6.61 (m, 6H, Ar-H), 3.49–3.41 (m, 4H, -C**H_2_**-N), 3.20–3.08 (m, 4H, -C**H_2_**-N). ^13^C NMR (100 MHz, DMSO-*d*_6_) *δ*_C_ 168.4 (C=O), 154.2, 151.4 (d, *J* = 256.1 Hz), 150.6, 140.0 (-C=N), 138.1, 132.0, 129.1, 125.4, 122.3, 119.8, 116.6, 116.4, 115.2, 113.9, 50.3 (piperazine-CH_2_), 47.8 (piperazine-CH_2_). HRMS-ESI (*m*/*z*): Calcd. for C_23_H_23_FN_5_O (M+H)^+^: 404.1887; Found: 404.1885.

***N*′-(4-(4-(4-Chlorophenyl)piperazin-1-yl)benzylidene)isonicotinohydrazide (IP3)**

Yellow solid, yield 48%, mp: 292–293 °C, Rf = 0.75 (MeOH/EtOAc 8:2). ^1^H NMR (400 MHz, DMSO-*d*_6_) *δ_H_* 11.88 (s, 1H, -NH, D_2_O exchangeable), 8.78 (bs, 2H, Ar-H), 8.35 (s, 1H, =CH), 7.81 (d, *J* = 4.2 Hz, 2H, Ar-H), 7.62 (d, *J* = 8.3 Hz, 2H, Ar-H), 7.26 (d, *J* = 8.2 Hz, 2H, Ar-H), 7.07 (d, *J* = 8.7 Hz, 2H, Ar-H), 7.02 (d, *J* = 8.2 Hz, 2H, Ar-H), 3.41 (bs, 4H, -C**H_2_**-N), 3.29 (bs, 4H, -C**H_2_**-N). ^13^C NMR (100 MHz, DMSO-*d*_6_) *δ*_C_ 161.6 (C=O), 152.5, 150.7, 150.1, 149.8, 141.2 (-C=N), 129.1, 129.0, 124.4, 123.1, 122.0, 117.6, 115.2, 48.4 (piperazine-CH), 47.5 (piperazine-CH). HRMS-ESI (*m*/*z*): Calcd. for C_23_H_23_ClN_5_O (M+H)^+^: 420.1591; Found: 420.1586.

***N*′-(4-(4-(4-Bromophenyl)piperazin-1-yl)benzylidene)isonicotinohydrazide (IP4)**

Yellow solid, yield 50%, mp: 278–279 °C, Rf = 0.72 (MeOH/EtOAc 9:1). ^1^H NMR (400 MHz, DMSO-*d*_6_) *δ*_H_ 11.87 (s, 1H, -NH, D_2_O exchangeable), 8.77 (d, *J* = 5.4 Hz, 2H, Ar-H), 8.35 (s, 1H, =CH), 7.81 (d, *J* = 5.6 Hz, 2H, Ar-H), 7.62 (d, *J* = 8.6 Hz, 2H, Ar-H), 7.37 (d, *J* = 8.8 Hz, 2H, Ar-H), 7.07 (d, *J* = 8.7 Hz, 2H, Ar-H), 6.96 (d, *J* = 8.9 Hz, 2H, Ar-H), 3.41 (bs, 4H, -C**H_2_**-N), 3.29 (bs, 4H, -C**H_2_**-N). ^13^C NMR (100 MHz, DMSO-*d*_6_) *δ*_C_ 161.7 (C=O), 152.5, 150.7, 150.4, 149.8, 141.2 (-C=N), 132.0, 129.1, 124.5, 122.0, 118.0, 115.2, 110.8, 48.2 (piperazine-CH), 47.4 (piperazine-CH). HRMS-ESI (*m*/*z*): Calcd. for C_23_H_23_BrN_5_O (M+H)^+^: 464.1086; Found: 464.1083.

***N*′-(4-(4-(2,4-Dimethylphenyl)piperazin-1-yl)benzylidene)isonicotinohydrazide (IP5)**

Yellow solid, yield 62%, mp: 263–264 °C, Rf = 0.78 (MeOH/EtOAc 8:2). ^1^H NMR (400 MHz, DMSO-*d*_6_) *δ*_H_ 11.90 (s, 1H, -NH, D_2_O exchangeable), 8.79 (d, *J* = 4.7 Hz, 2H, Ar-H), 8.35 (s, 1H, =CH), 7.85 (d, *J* = 4.5 Hz, 2H, Ar-H), 7.62 (d, *J* = 8.3 Hz, 2H, Ar-H), 7.06 (d, *J* = 9.0 Hz, 2H, Ar-H), 7.05–6.91 (m, 3H, Ar-H), 2.95 (s, 4H, -C**H_2_**-N), 2.25 (s, 3H, -C**H_3_**), 2.21 (s, 3H, -C**H_3_**), (remaining piperazine signal overlapped with solvent). ^13^C NMR (100 MHz, DMSO-*d*_6_) *δ*_C_ 161.5 (C=O), 152.8, 150.1, 150.0, 148.9, 141.8 (-C=N), 132.5, 132.1, 132.0, 129.1, 127.5, 124.2, 122.3, 119.1, 115.0, 51.9 (piperazine-C), 48.2 (piperazine-C), 20.8 (-CH_3_), 17.9 (-CH_3_). HRMS-ESI (*m*/*z*): Calcd. for C_25_H_28_N_5_O (M+H)^+^: 414.2294; Found: 414.2296.

***N*′-(4-(4-(4-(Trifluoromethyl)phenyl)piperazin-1-yl)benzylidene)isonicotinohydrazide (IP6)**

Yellow solid, yield 47%, mp: 302–303 °C, Rf = 0.6 (MeOH/EtOAc 9:1). ^1^H NMR (400 MHz, DMSO) *δ*_H_ 11.83 (s, 1H, -NH, D_2_O exchangeable), 9.05–8.55 (m, 2H, Ar-H), 8.36 (s, 1H, =CH), 8.05–7.75 (m, 2H, Ar-H), 7.62 (d, *J* = 8.8 Hz, 2H, Ar-H), 7.53 (d, *J* = 8.5 Hz, 2H, Ar-H), 7.12 (d, *J* = 8.6 Hz, 2H, Ar-H), 7.06 (d, *J* = 8.5 Hz, 2H, Ar-H), 3.45 (s, 8H, -C**H_2_**-N). ^13^C NMR (100 MHz, DMSO-*d*_6_) *δ*_C_ 161.9 (C=O), 153.4, 152.5, 150.7, 149.9, 141.3 (-C=N), 129.1, 126.7, 124.3, 122.0, 118.3, 115.2, 115.1, 114.7, 47.2 (piperazine-C), 47.1 (piperazine-C). HRMS-ESI (*m*/*z*): Calcd. for C_24_H_23_F_3_N_5_O (M+H)^+^: 454.1855; Found: 454.1848.

***N*′-(4-(4-(4-Methoxyphenyl)piperazin-1-yl)benzylidene)isonicotinohydrazide (IP7)**

Cream solid, yield 57%, mp: 284–285 °C, Rf = 0.66 (MeOH/EtOAc 8:2). ^1^H NMR (400 MHz, DMSO-*d*_6_) *δ*_H_ 11.84 (s, 1H, -NH, D_2_O exchangeable), 8.77 (d, *J* = 4.5 Hz, 2H), 8.35 (s, 1H, =CH), 7.81 (d, *J* = 4.4 Hz, 2H, Ar-H), 7.62 (d, *J* = 8.1 Hz, 2H, Ar-H), 7.07 (d, *J* = 8.4 Hz, 2H, Ar-H), 6.96 (d, *J* = 8.7 Hz,2H, Ar-H), 6.85 (d, *J* = 8.7 Hz, 2H, Ar-H), 3.69 (s, 3H, -OCH_3_), 3.41 (bs, 4H, -C**H_2_**-N), 3.20–3.04 (m, 4H, -C**H_2_**-N). ^13^C NMR (100 MHz, DMSO-*d*_6_) *δ*_C_ 161.3 (C=O), 153.6, 152.2, 150.2, 149.5, 141.0 (-C=N), 128.7, 124.1, 122.2, 122.0, 118.5, 115.2, 114.8, 55.3 (-OCH_3_), 50.0 (piperazine-C), 47.3 (piperazine-C). HRMS-ESI (*m*/*z*): Calcd. for C_24_H_26_N_5_O_2_ (M+H)^+^: 416.2087; Found: 416.2084.

***N*′-(4-(4-(Pyridin-2-yl)piperazin-1-yl)benzylidene)isonicotinohydrazide (IP8)**

Yellow solid, yield 73%, mp: 289–290 °C, Rf = 0.37 (MeOH/EtOAc 9:1). ^1^H NMR (400 MHz, DMSO-*d*_6_) *δ*_H_ 11.85 (s, 1H, -NH, D_2_O exchangeable), 8.77 (d, *J* = 6.0 Hz, 2H, Ar-H), 8.35 (s, 1H, =CH), 8.25–8.10 (m, 1H, Ar-H), 7.81 (dd, *J* = 4.5, 1.6 Hz, 2H, Ar-H), 7.75–7.41 (m, 3H, Ar-H), 7.06 (d, *J* = 8.9 Hz, 2H, Ar-H), 6.89 (d, *J* = 8.6 Hz, 1H, Ar-H), 6.67 (dd, *J* = 7.0, 4.9 Hz, 1H, Ar-H), 3.83–3.53 (m, 4H, -C**H_2_**-N), 3.51–3.36 (m, 4H, -C**H_2_**-N). ^13^C NMR (100 MHz, DMSO-*d*_6_) *δ*_C_ 161.7 (C=O), 159.3, 152.6, 150.7, 149.8, 148.1, 141.2 (-C=N), 138.1, 129.0, 124.3, 121.9, 115.1, 113.7, 107.7, 47.4 (piperazine-C), 44.8 (piperazine-C). HRMS-ESI (*m*/*z*): Calcd. for C_22_H_23_N_6_O (M+H)^+^: 387.1933; Found: 387.1934.

***N*′-(4-(4-(Pyrimidin-2-yl)piperazin-1-yl)benzylidene)isonicotinohydrazide (IP9)**

Yellow solid, yield 32%, mp: 288–289.5 °C, Rf = 0.64 (MeOH/EtOAc 7:3). ^1^H NMR (400 MHz, DMSO-*d*_6_) *δ*_H_ 11.98 (s, 1H, -NH, D_2_O exchangeable), 8.82 (s, 2H, Ar-H), 8.48–8.32 (m, 3H, =CH and Ar-H), 7.91 (s, 2H, Ar-H), 7.62 (d, *J* = 7.4 Hz, 2H, Ar-H), 7.06 (d, *J* = 7.7 Hz, 2H, Ar-H), 6.68 (d, *J* = 3.4 Hz, 1H, Ar-H), 3.88 (bs, 4H, -C**H_2_**-N), 3.38 (bs, 4H, -C**H_2_**-N). ^13^C NMR (100 MHz, DMSO-*d*_6_) *δ*_C_ 161.5 (C=O), 161.4, 158.5, 152.6, 150.0, 149.8, 142.2 (-C=N), 129.1, 124.3, 122.5, 115.2, 110.9, 47.4 (piperazine-C), 43.4 (piperazine-C). HRMS-ESI (*m*/*z*): Calcd. for C_21_H_22_N_7_O (M+H)^+^: 388.1886; Found: 388.1888.

***N*′-(4-(4-Methylpiperazin-1-yl)benzylidene)isonicotinohydrazide (IP10)**

Yellow solid, yield 60%, mp: 238–239.5 °C, Rf = 0.15 (MeOH/EtOAc 7:3). ^1^H NMR (400 MHz, DMSO-*d*_6_) *δ*_H_ 11.86 (s, 1H, -NH, D_2_O exchangeable), 9.38–8.59 (m, 2H, Ar-H), 8.33 (s, 1H, =CH), 7.81 (s, 2H, Ar-H), 7.58 (d, *J* = 8.7 Hz, 2H, Ar-H), 6.99 (d, *J* = 8.7 Hz, 2H, Ar-H), 3.33–3.18 (m, 4H, -C**H_2_**-N), 2.52–2.38 (m, 4H, -C**H_2_**-N), 2.21 (s, 3H, -C**H_3_**-). ^13^C NMR (100 MHz, DMSO-*d*_6_) *δ*_C_ 161.7 (C=O), 152.7, 150.7, 149.8, 141.2 (-C=N), 129.0, 124.1, 121.9, 114.9, 54.8 (piperazine-C), 47.4 (piperazine-C), 46.2 (-CH_3_). HRMS-ESI (*m*/*z*): Calcd. for C_18_H_22_N_5_O (M+H)^+^: 324.1824; Found: 324.1822.

***N*′-(4-(4-Cyclohexylpiperazin-1-yl)benzylidene)isonicotinohydrazide (IP11)**

Yellow solid, yield 44%, mp: 248–249 °C, Rf = 0.3 (MeOH/EtOAc 7:3). ^1^H NMR (400 MHz, DMSO-*d*_6_) *δ*_H_ 11.78 (s, 1H, -NH, D_2_O exchangeable), 8.76 (d, *J* = 4.6 Hz, 2H, Ar-H), 8.35 (s, 1H, =CH), 7.81 (d, *J* = 4.5 Hz, 2H, Ar-H), 7.57 (d, *J* = 8.3 Hz, 2H, Ar-H), 6.97 (d, *J* = 8.3 Hz, 2H, Ar-H), 3.23 (s, 4H, -C**H_2_**-N), 2.62 (s, 4H, -C**H_2_**-N), 2.34–2.08 (m, 1H, cyclohexyl-H), 1.88–1.62 (m, 4H, cyclohexyl-H), 1.63–1.51 (m, 1H, cyclohexyl-H), 1.39–0.89 (m, 5H, cyclohexyl-H). ^13^C NMR (100 MHz, DMSO-*d*_6_) *δ*_C_ 161.7 (C=O), 152.6, 150.7, 149.9, 141.1 (-C=N), 129.0, 124.1, 121.9, 114.9, 63.2 (cyclohexyl-C), 48.7 (piperazine-C), 47.7 (piperazine-CH_2_), 28.5 (cyclohexyl-C), 26.2 (cyclohexyl-C), 25.6 (cyclohexyl-C). HRMS-ESI (*m*/*z*): Calcd. for C_23_H_30_N_5_O (M+H)^+^: 392.2450; Found: 392.2449.

**Ethyl 1-(4-((2-isonicotinoylhydrazono)methyl)phenyl)piperidine-4-carboxylate (IP12)**

Mustard yellow solid, yield 88%, mp: 226–227 °C, Rf = 0.75 (MeOH/EtOAc 7:3). ^1^H NMR (400 MHz, DMSO-*d*_6_) *δ*_H_ 11.82 (s, 1H, -NH, D_2_O exchangeable), 8.77 (d, *J* = 4.5 Hz, 2H, Ar-H), 8.33 (s, 1H, =CH), 7.81 (d, *J* = 4.5 Hz, 2H, Ar-H), 7.57 (d, *J* = 8.4 Hz, 2H, Ar-H), 7.00 (d, *J* = 8.5 Hz, 2H, Ar-H), 4.08 (q, *J* = 6.9 Hz, 2H, -CH_2_), 3.79 (d, *J* = 12.6 Hz, 2H, piperidine-H), 3.03–2.83 (m, 2H, piperidine-H), 2.62–2.54 (m, 1H, piperidine-H), 2.05–1.86 (m, 2H, piperidine-H), 1.72–1.55 (m, 2H, piperidine-H), 1.19 (t, *J* = 7.0 Hz, 3H, -CH_3_). ^13^C NMR (100 MHz, DMSO) *δ*_C_ 174.1 (C=O), 161.2 (C=O), 152.1, 150.3, 149.4, 140.8 (-C=N), 128.6, 123.3, 121.5, 114.8, 59.9 (-CH_2_), 46.8 (piperidine-C), 40.1 (piperidine-C), 27.2 (piperidine-C), 14.1 (-CH_3_). HRMS-ESI (*m*/*z*): Calcd. for C_21_H_25_N_4_O_3_ (M+H)^+^: 381.1927; Found: 381.1928.

**1-(4-((2-Isonicotinoylhydrazono)methyl)phenyl)piperidine-4-carboxamide (IP13)**

Yellow solid, yield 51%, mp: 329–330 °C, Rf = 0.31 (MeOH/EtOAc 8:2). ^1^H NMR (400 MHz, DMSO-*d*_6_) *δ*_H_ 11.81 (s, 1H, -NH, D_2_O exchangeable), 8.77 (d, *J* = 5.6 Hz, 2H, Ar-H), 8.33 (s, 1H, =CH), 7.80 (d, *J* = 5.7 Hz, 2H, Ar-H), 7.57 (d, *J* = 8.6 Hz, 2H, Ar-H), 7.29 (s, 1H, -NH_2,_, D_2_O exchangeable), 7.00 (d, *J* = 8.7 Hz, 2H, Ar-H), 6.78 (s, 1H, -NH_2_, D_2_O exchangeable), 3.87 (d, *J* = 12.8 Hz, 2H, piperidine-H), 2.82 (q, *J* = 12.2, 11.1 Hz, 2H, piperidine-H), 2.46–2.21 (m, 1H, piperidine-H), 1.87–1.73 (m, 2H, piperidine-H), 1.72–1.53 (m, 2H, piperidine-H). ^13^C NMR (100 MHz, DMSO) *δ*_C_ 176.2 (C=O), 161.2 (C=O), 152.2, 150.3, 149.4, 140.8 (-C=N), 128.6, 123.14, 121.5, 114.6, 47.2 (piperidine-C), 41.5 (piperidine-C), 27.7 (piperidine-C). HRMS-ESI (*m*/*z*): Calcd. for C_19_H_22_N_5_O_2_ (M+H)^+^: 352.1773; Found: 352.1773.

### 2.2. Evaluation of Antitubercular Activity and Cytotoxicity

#### 2.2.1. Microplate Alamar Blue Assay (MABA) Protocol for Antimycobacterial Testing

The *Mtb* drug-sensitive reference strain H37Rv and drug-resistant clinical isolates were inoculated onto 7H10+OADC agar plates and incubated at 37 °C. From these agar plates, pure colonies were transferred to OADC-enriched liquid Middlebrook 7H9 medium, allowing them to grow to the mid-logarithmic phase. Subsequently, the *Mtb* cultures were further expanded and inoculated into 96-well plates containing Middlebrook 7H9 medium at increasing concentrations of the test compounds, targeting approximately 4 × 10^5^ CFU/mL in 200 μL per well. Following a one-week incubation at 37 °C, each well was supplemented with 32.5 μL of a resazurin-tween mixture (in an 8:5 ratio of 0.6 mM Resazurin in 1X PBS and 20% Tween 80). The resulting fluorescent resorufin produced during this process is utilized to determine the minimum inhibitory concentration (MIC) of the compounds under investigation [19].

#### 2.2.2. Assay for Host Cytotoxicity Determination

The MTT (3-(4,5-dimethylthiazol-2-yl)-2,5-diphenyltetrazolium bromide) assay was applied to determine the cytotoxicity of the synthesized molecules against Human Embryonic Kidney cells (HEK 293) and adenocarcinomic human alveolar basal epithelial cells (A549) cell lines, obtained from the American Type Culture Collection (ATCC) with the accession numbers ATCC CRL-1573 and ATCC CCL-185, respectively. Compounds that demonstrated significant antimycobacterial activity (**IP8**–**IP13**) were dissolved in DMSO to prepare stock solutions. These stocks were further diluted in culture medium to achieve the desired concentrations, ensuring the final DMSO concentration did not exceed 1% (*v*/*v*) in any well. A vehicle control containing 1% DMSO in culture medium without any compound was included to account for any solvent-related effects. Cells were seeded at a density of 1 × 10^4^ cells per well in a sterile 96-well microtiter plate and incubated for 24 h to allow attachment. Following this, the compounds were added in ten-fold serial dilutions ranging from 100 μM to 0.001 μM, and the plates were incubated for 48 h at 37 °C with 5% CO_2_.

After incubation, the medium was removed and replaced with 10 μL of MTT solution (5 mg/mL) in each well, followed by a 3 h incubation under the same conditions. The MTT reagent was then discarded, and 100 μL of DMSO was added to each well to solubilize the formazan crystals. Absorbance was measured at 580 nm using a Thermo Scientific Varioskan LUX microplate reader, with blanks subtracted from each value. All experiments were performed in three independent biological replicates, for every concentration tested. The IC_50_ values were calculated using GraphPad Prism 10.6.0 [28,29].

### 2.3. InhA Inhibition

InhA was produced and purified according to a previously reported procedure [25]. Inhibitor activity assays were performed by monitoring NADH concentration at 340 nm using a Cary 300 UV-vis spectrophotometer (Agilent, Santa Clara, CA, USA). Following the protocol described previously [30], 2-*trans*-dodecenoyl-CoA (DDCoA) was employed as a substrate analogue. Specifically, 840 μL of buffer (30 mM PIPES, 150 mM NaCl, pH 6.8) was added in a 1 mL cuvette, followed by the addition of an inhibitor dissolved in DMSO, ensuring a final DMSO concentration of 5% *v*/*v* (50 μL). Then, 50 μL of 1 mM DDCoA stock solution in buffer and 50 μL of 5 mM NADH (Alfa Aesar, Haverhill, MA, USA) stock solution in buffer were added. The mixture was pre-incubated for 1 min at 25 °C before initiating the reaction, with the addition of 10 μL of His6-InhA from a 5 μM stock solution, as previously described. The rate of NADH oxidation was monitored both in the presence and absence of the inhibitor. The inhibitory potency of the compounds was expressed as the percentage inhibition of His6-InhA activity relative to the control reaction conducted without an inhibitor. All activity assays were carried out in duplicate or triplicate trials.

### 2.4. Molecular Modeling Studies

#### 2.4.1. Molecular Docking and Molecular Dynamics Simulations

The chemical structure of the most effective InhA inhibitor, **IP11**, was drawn and docked into the binding pocket of InhA enzyme (PDB Code: 1P44) [31] in the presence of the cofactor nicotinamide adenine dinucleotide (NAD) with the default parameters of AutoDock [32], implemented in LigandScout 4.5 [33]. The binding poses obtained were inspected visually and the figures reporting the molecular docking data were prepared using LigandScout.

For molecular dynamics simulations (MD), the protein-ligand complex was set up using the chosen binding pose of **IP11** in the InhA binding site in Maestro 13.1 [34]. The complex was centered inside an orthorhombic box with a 10 Å space preserved between the protein surface and the edges of the box. The TIP3P water model was then used to fill the box, and sodium chloride was added to achieve a physiological concentration of 0.15 M. MD simulations of the prepared protein-ligand complex were performed using Desmond (v2022.01) software [35] at the Molecular Design Laboratory, Freie Universität Berlin, utilizing graphics processing units (GPUs). The simulations were performed at 300 K and a pressure of 1.01325 bar for 50 ns, yielding 1000 conformational snapshots of the protein–ligand complexes. The resulting trajectories and conformations were analyzed using the Visual Molecular Dynamics (VMD) software (v.1.9.3) [36]. Subsequently, the dynophore application was employed to examine the dynamic interactions between the proteins and ligands [37]. This approach combines three-dimensional pharmacophore modeling with molecular dynamics to identify interaction sites within each frame of the simulation. The dynophore representations, depicted as point clouds, illustrate the density of interaction types, which helps clarify the binding interaction patterns.

#### 2.4.2. Prediction of Drug-Likeness

The chemical structures of **IP1**–**IP13** were drawn, and their key parameters were forecasted employing the online SwissADME tool (http://www.swissadme.ch) [38].

## 3. Results and Discussion

### 3.1. Chemistry

**Scheme 1:**
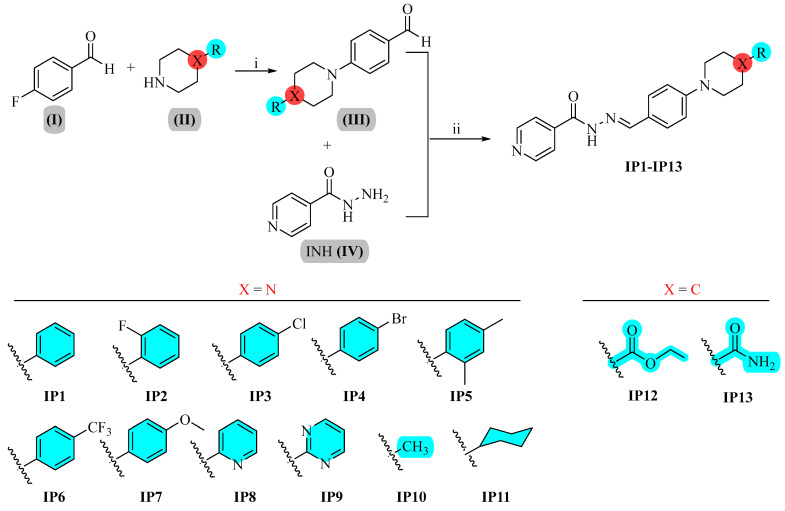
General synthetic pathway for compounds **IP1**–**IP13.** Reagents and conditions: (**i**) K_2_CO_3_, DMF, reflux; (**ii**) EtOH, reflux.

The synthetic routes for the target INH-piperazine/piperidine derivatives (**IP1**–**IP13**) were outlined in Scheme 1. The key intermediates, piperidine/piperazine-substituted benzaldehydes (**III**), were synthesized by reacting *p*-fluorobenzaldehyde (**I**) with the corresponding amines (piperazine and piperidine derivatives) (**II**) in DMF under reflux conditions with potassium carbonate as a base. The resulting 4-aminobenzaldehyde derivatives (**III)** were then reacted with INH (**IV**) in an alcoholic medium, yielding the corresponding hydrazones (**IP1**–**IP13**) with yields ranging from 32% to 88% (Scheme 1).

The chemical structures of the final products were determined using ^1^H NMR, ^13^C NMR, and mass spectral data. In the ^1^H NMR spectra (recorded in DMSO-*d*_6_) of the synthesized *N*-acylhydrazones (**IP1**–**IP13**), the characteristic singlet peak for the imine N=CH protons appeared in the expected range of *δ* 8.50–8.07 ppm. Additionally, amide inversion caused the CONH protons to appear as a broad singlet within the range of *δ* 12.10–11.77 ppm. In the ^13^C NMR spectra, the carbonyl carbon showed peaks in the range of 169–170 ppm, while the azomethine N=CH carbon exhibited peaks between 146 and 149 ppm.

The ^1^H NMR spectra of the synthesized *N*-acylhydrazones (**IP1**–**IP13**) exhibited a single set of signals corresponding to the amide and imine protons, which confirms the predominant formation of the (*E*)-isomer. This observation can be attributed to the steric hindrance present around the C=N bond, aligning with previously reported free energy calculations and literature that suggest aromatic *N*-acylhydrazones maintain a planar structure and mainly exist in the (*E*)-configuration [39].

### 3.2. Evaluation of Antimycobacterial Activity and Cytotoxicity

We evaluated the antitubercular activity of all synthesized target molecules (**IP1**–**IP13**) on *Mtb* H37Rv. We further screened the most promising compounds, with the MIC values ≤ 0.78 μM on *Mtb* H37Rv, against two INH-resistant clinical isolates with *katG* or *inhA* promoter mutations by applying the Microplate Alamar Blue Assay (MABA) protocol. Given that toxicity poses a considerable challenge to the development of new antitubercular agents, we further evaluated the cytotoxic effects of the most effective compounds against *Mtb* strains on HEK 293 and A549 cell lines. Table 1 presents the minimum inhibitory concentration (MIC) values for three tested *Mtb* strains and toxicity data for **IP1**–**IP13**.

**Figure 5:**
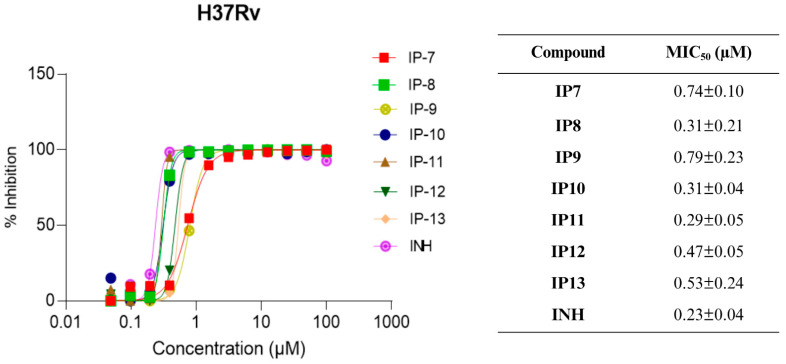
MIC_50_ values of **IP7**–**IP13** and INH against *Mtb* H37Rv.

According to the MIC values obtained, **IP7**-**IP13** demonstrated MIC values ≤ 0.78 μM against *Mtb* H37Rv; therefore, we also calculated MIC_50_ values of these derivatives (Figure 5).

All compounds inhibited the growth of *Mtb* H37Rv with MIC values ranging from 0.39 to 100 μM, except for **IP2** with a MIC value of >100 μM. Among the molecules with MIC values of ≤0.78 μM, **IP11** exhibited the lowest MIC_50_ value (0.29 μM) against *Mtb* H37Rv in this series. MIC_50_ values of the most promising other compounds (**IP7**–**IP10**, **IP12**, and **IP13**) ranged between 0.31 and 0.79 μM. Subsequently, **IP7**–**IP13** were further tested on two INH-resistant (*inhA* promoter mutant and *katG* S315T mutant) clinical isolates. As anticipated, the MIC values of INH against these INH-resistant clinical isolates were higher than the MIC value observed for the INH-sensitive *Mtb* H37Rv. Against the *inhA* promotor mutant INH-resistant *Mtb* clinical isolate, **IP11** demonstrated a lower MIC value (0.78 μM) compared to INH (1.56 μM). Additionally, **IP8**, **IP10**, **IP12**, and **IP13** exhibited the same MIC value (1.56 μM) as observed for INH. **IP7**–**IP13** were less effective against INH-resistant *Mtb* isolate with *katG* mutation. Interestingly, **IP11** was again the most active antimycobacterial agent with a MIC value of 12.5 μM on this isolate. **IP11** was followed by **IP9**, **IP10**, and **IP12** with their MIC values found to be 25 μM.

When we correlated the antitubercular data with the chemical structures of the compounds, it was observed that **IP11** with cyclohexyl ring on the piperazine moiety was the most attractive molecule in this series demonstrating the lowest MIC values against all tested *Mtb* strains. Except for **IP7** carrying methoxy group on the phenyl ring with a MIC value of 0.78 μM, phenyl ring with lipophilic substituents on the piperazine moiety (**IP1**–**IP6**) was not a preferential modification on these compounds for antitubercular activity. On the other hand, when the phenyl ring was replaced with less lipophilic rings (**IP8** and **IP9**) or alkyl groups (**IP10** and **IP11**), antimycobacterial activity of the piperazine derivatives significantly increased. Among the compounds carrying piperazine ring, the most preferential substitution pattern was to introduce cyclohexyl moiety (**IP11**) onto 4th position of the ring to yield the most effective antimycobacterial agent against all *Mtb* strains tested. Although we have only two piperidine-featuring compounds (**IP12** and **IP13**), they are among the most attractive derivatives in this series. It is noteworthy that these compounds also have less lipophilic substituents, namely ethylcarboxylate (**IP12**) and caboxamide (**IP13**) on the piperidine moiety compared to halogen-carrying piperazine derivatives.

Additionally, we screened the compounds that demonstrated significant antimycobacterial effect on all *Mtb* strains tested (**IP8**–**IP13**) for their host cytotoxicity against a healthy cell line, HEK 293. Additionally, the compounds were evaluated using the human alveolar epithelial type 2 carcinoma cell line A549, given that the lung epithelium represents the primary lung surface that contacts with *Mtb* [40]. According to the data obtained, all molecules tested presented IC_50_ values of >100 μM, which means the most bioactive compounds in this study were found to be non-toxic, even at higher concentrations than those required for effective TB treatment. Consequently, we identified new antitubercular agents with favorable activity and toxicity profiles.

### 3.3. InhA Inhibition

**Figure 6:**
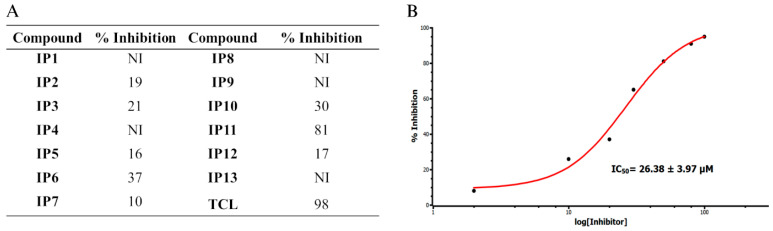
InhA inhibition values of **IP1**–**IP13** and TCL (triclosan) at 50 μM concentration (**A**), IC_50_ determination for **IP11** against InhA enzyme (**B**). NI: No inhibition.

INH primarily inhibits the InhA enzyme, which plays a critical role in the biosynthesis of long-chain fatty acids in *Mtb*. These fatty acids, particularly mycolic acids, are essential structural elements of the bacterial cell wall and are necessary for the survival of *Mtb* [7]. Upon its activation by KatG, INH produces a reactive isonicotinoyl radical intermediate, which promptly forms a covalent bond with the NADH, cofactor of InhA. Mutations in the *katG* gene reduce the activation of INH, leading to the predominant mechanism of resistance against INH [41]. Consequently, developing direct InhA inhibitors that do not need activation by KatG is seen as a logical way to address this resistance issue [42]. Therefore, we evaluated our INH-derived molecules against InhA to explore whether their potential antimycobacterial activity mechanism involves the direct inhibition of the enzyme (Figure 6).

Based on the inhibition values obtained against InhA, all compounds except for **IP11**, failed to effectively inhibit the enzyme. This means that the structural modifications of the compounds may lead to a change in their protein target profile. Interestingly, **IP11** exhibited 81% inhibition at a concentration of 50 μM with an IC_50_ value of 26.38 ± 3.97 μM. Since the molecules differ primarily in the substituents on their piperidine or piperazine moiety, the presence of a bulky cyclohexyl group appears to be favorable for InhA inhibition.

### 3.4. Molecular Modeling Studies

#### 3.4.1. Molecular Docking and Molecular Dynamics Simulations

**Figure 7:**
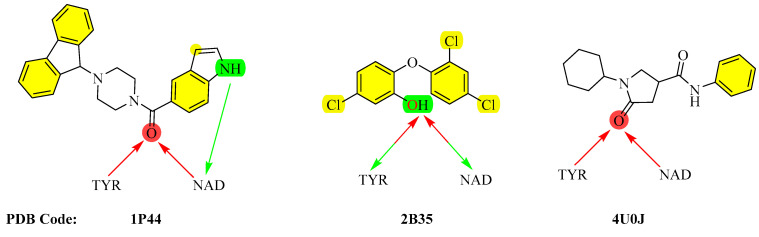
Chemical structures of selected inhibitors co-crystallized within InhA. Parts of the compounds involved in binding to InhA are highlighted. Pharmacophores are defined as follows: green arrow-hydrogen bond donor, red arrow-hydrogen bond acceptor, yellow area-hydrophobic contact.

The InhA active pocket is sufficiently large to accommodate a variety of direct inhibitors with diverse chemical structures, including triclosan and phenoxy-phenol derivatives, aryl carboxamides, oxopyrrolidine-3-carboxamides, and hydroxypyridones [43]. Despite this flexibility, direct inhibitors generally block InhA by forming two critical hydrogen bonds with tyrosine and the nicotinamide adenine dinucleotide (NAD) cofactor of the enzyme in the catalytic site of InhA. Further hydrophobic contacts are necessary within the lipophilic regions of the InhA binding pocket [7] (Figure 7).

**Figure 8:**
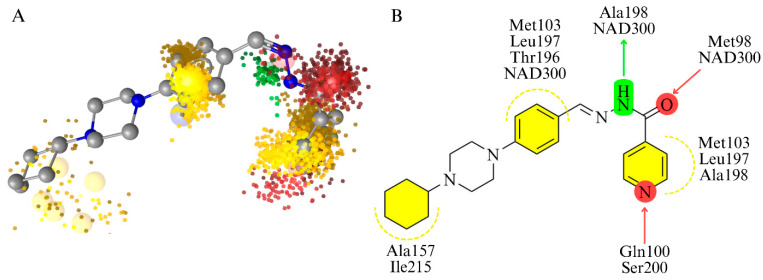
Dynophore of **IP11** with interacting amino acid residues in InhA (**A**) and 2D-representation of the dynophores (**B**) Hydrophobic interactions (yellow clouds and circles), hydrogen bond donor (green clouds and arrow), hydrogen bond acceptor (red clouds and arrows).

We first conducted molecular docking studies to describe the binding mode of **IP11**, the only effective InhA inhibitor in this series. Subsequently, we utilized MD simulations in conjunction with the dynophore methodology to analyze the interaction dynamics and evaluate the stability of **IP11** within InhA (Figure 8).

According to the data obtained, **IP11** was found to be highly stable in the binding pocket of InhA. Pyridine moiety of INH as well as phenyl and cyclohexyl rings of **IP11** formed hydrophobic contacts with various amino acid residues. It is worth mentioning that the phenyl ring engaged in hydrophobic interaction with the cofactor NAD^+^. **IP11** was stabilized in the active site of INH also through forming hydrogen bonds. The nitrogen atom of the pyridine ring acted as hydrogen bond acceptor for Gln100 and Ser200. Additionally, the oxygen atom of the carbonyl group of the hydrazone functionality formed hydrogen bonds with Met98 and NAD300. N-H group of hydrazone moiety was a hydrogen bond donor for hydrogen bonding with Ala198 and NAD300. As previously mentioned, hydrogen bonds generally with the co-factor NAD and the tyrosine residue are required for InhA inhibition in the catalytic site. Although **IP11** does not form any interaction with Tyr158, the ligand is highly stabilized in InhA through the hydrogen bonds with NAD and other additional hydrogen bonds. Moreover, hydrophobic contacts via terminal rings of InhA inhibitors are also characteristic interactions for inhibiting InhA. **IP11** formed lipophilic interactions via its terminal pyridine and cyclohexyl moieties. Consequently, the interaction dynamics of **IP11**, the only effective InhA inhibitor in this series, could shed light on the design of new antitubercular agents demonstrating their effect via InhA inhibition.

#### 3.4.2. Prediction of Drug-Likeness

Unsuitable physicochemical properties of drug candidates pose a significant challenge in transforming bioactive compounds into drugs during the drug development process. Consequently, we conducted in silico calculations to determine the drug-like characteristics of our compounds. We began by calculating the descriptors that form Lipinski’s rule of five, which assesses whether a chemical compound with a desirable biological profile has the appropriate physicochemical properties to be orally active in humans. To demonstrate oral bioavailability, a compound should comply with the following criteria: molecular weight (MW) of 500 or less, n-octanol/water partition coefficient (log P) of no more than 5, maximum of 5 hydrogen bond donors (HBD), and no more than 10 hydrogen bond acceptors (HBA). In addition, we anticipated the topological polar surface areas (TPSAs) and the counts of rotatable bonds (NRBs) of the compounds, as both are considered essential factors in forecasting the oral bioavailability of new molecular entities. The calculated key parameters of **IP1**–**IP13** are presented in Table 2.

According to the estimated parameters, all compounds fully comply with Lipinski’s rule of five. Furthermore, the computed LogP values indicate that the compounds exhibit a higher lipophilicity than INH (LogP = −0.35, as calculated in SwissADME). In addition to mutations occurring in either the *katG* gene or the *inhA* promoter, the low hydrophobicity of INH is associated with the prolonged duration of TB treatment. The outer layer of *Mtb*, composed of mycolic acids, exhibits a high degree of lipophilicity, which inhibits the efficient penetration of INH. Additionally, the hydrophilic nature of INH contributes to its low permeability within *Mtb* granulomas. Consequently, this study focuses on augmenting the lipophilic properties of INH through chemical modifications to improve its diffusion capability across the cell membrane. The compounds exhibiting exceptional antitubercular activity (MIC ≤ 0.78 μM) in this series possess a LogP value of less than 3. This indicates that while enhancing lipophilicity is essential, it should remain below this threshold for these molecules to achieve the optimal antimycobacterial effect.

The count of rotatable bonds, a critical indicator of a molecule’s conformational flexibility, is ideally maintained below ten to reduce the extent of conformational variability during interactions with biological targets. **IP1**–**IP13** adhere to this guideline, as each contains fewer rotatable bonds. Furthermore, the polar surface area (TPSA) of these compounds ranges from 60.83 to 100.68 Å^2^. Most clinically utilized drug molecules typically possess a TPSA below 140–150 Å^2^, indicating that our compounds fall within an acceptable range. As a result, the physicochemical characteristics of our compounds indicate their significant potential as promising candidates in developing new antimycobacterial drugs.

### 3.5. Structure-Activity Relationships

**Figure 9:**
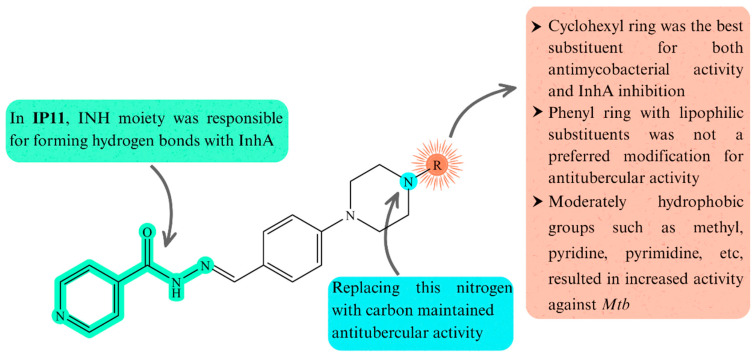
Structure-activity relationships of INH-based hydrazones with antitubercular potential.

To establish the structure-activity relationships of INH-based hydrazones with antimycobacterial activity, we analyzed the chemical structures of **IP1**–**IP13** along with the data obtained against *Mtb* strains, from InhA inhibition and molecular modeling studies. Our main findings are summarized in Figure 9.

## 4. Conclusions

The swift rise and worldwide spread of drug-resistant and MDR *Mtb* strains have once again highlighted TB as a pressing public health issue globally. In particular, resistance to INH, one of the frontline drugs used in TB treatment, represents a significant obstacle to the effective management and reduction of the worldwide incidence of the disease. In this study, we explored the chemical modification of the cost-effective drug INH to establish a novel pathway for developing new antimycobacterial agents. To this end, we synthesized a series of novel INH derivatives by attaching this drug to piperidine or piperazine rings through a hydrazone linkage (**IP1**–**IP13**). Data collected from MABA and MTT assays regarding antitubercular activity and toxicity, respectively, supported the efficacy of these compounds for the treatment of TB. Evaluation of their inhibitory effect on InhA, the main target of INH, indicated that the antimycobacterial activity of our compounds, except for **IP11**, was not primarily due to direct InhA inhibition. The binding mode of **IP11** to InhA was further investigated through molecular docking and molecular dynamics simulations. Additionally, the calculation of key physicochemical parameters confirmed the drug-likeness of the compounds and highlighted the role of moderately increased lipophilicity in improving the biological profile of **IP1**–**IP13** relative to INH. Overall, this study presents a novel approach to modifying INH in an effort to address the significant issue of drug resistance in TB treatment.

## Figures and Tables

**Table 1:** MIC values against *Mtb* H37Rv and INH-resistant strains, and cytotoxicity data of **IP1**–**IP13**.

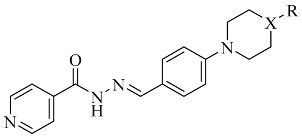							
			MIC (μM)			Toxicity IC_50_ (μM) *	
Compound	X	R	H37Rv	*inhA* ^+(a)^	*katG* ^+(b)^	HEK 293	A549
**IP1**	N	phenyl	50	-	-	-	-
**IP2**	N	2-fluorophenyl	>100	-	-	-	-
**IP3**	N	4-chlorophenyl	50	-	-	-	-
**IP4**	N	4-bromophenyl	100	-	-	-	-
**IP5**	N	2,4-dimethylphenyl	50	-	-	-	-
**IP6**	N	4-trifluoromethylphenyl	12.5	-	-	-	-
**IP7**	N	4-methoxyphenyl	0.78	25	100	-	-
**IP8**	N	pyridin-2-yl	0.39	1.56	50	>100	>100
**IP9**	N	pyrimidin-2-yl	0.78	3.12	25	>100	>100
**IP10**	N	methyl	0.39	1.56	25	>100	>100
**IP11**	N	cyclohexyl	0.39	0.78	12.5	>100	>100
**IP12**	C	ethylcarboxylate	0.78	1.56	25	>100	>100
**IP13**	C	carboxamide	0.78	1.56	50	>100	>100
**INH**			0.39	1.56	6.25	-	-

^(a)^ INH-resistant *Mtb* clinical isolate with *inhA* promoter mutation. ^(b)^ INH-resistant *Mtb* clinical isolate with *katG* S315T mutation. * Experiments were performed in triplicate.

**Table 2:** Calculated descriptors and molecular properties of **IP1**–**IP13**.

Compound	MW ^a^	LogP ^b^	HBD ^c^	HBA ^d^	NRB ^e^	TPSA ^f^	Lipinski’s Violation
**IP1**	385.46	2.84	1	3	6	60.83	0
**IP2**	403.45	3.13	1	4	6	60.83	0
**IP3**	419.91	3.37	1	3	6	60.83	0
**IP4**	464.36	3.46	1	3	6	60.83	0
**IP5**	413.51	3.49	1	3	6	60.83	0
**IP6**	453.46	3.86	1	6	7	60.83	0
**IP7**	415.49	2.86	1	4	7	70.06	0
**IP8**	386.45	2.26	1	4	6	73.72	0
**IP9**	387.44	1.77	1	5	6	86.61	0
**IP10**	323.39	1.66	1	4	5	60.83	0
**IP11**	391.51	2.99	1	4	6	60.83	0
**IP12**	380.44	2.54	1	5	8	83.89	0
**IP13**	351.40	1.42	2	4	6	100.68	0

^a^ MW: molecular weight. ^b^ LogP: logarithm of *n*-octanol-water partition coefficient. ^c^ HBA: number of hydrogen bond acceptors. ^d^ HBD: number of hydrogen bond donors. ^e^ NRB: number of rotatable bonds. ^f^ TPSA: topological polar surface area.

## Data Availability

The original contributions presented in this study are included in the article/supplementary material. Further inquiries can be directed to the corresponding authors.

## Supplementary Materials

The following supporting information can be downloaded at: https://www.mdpi.com/article/10.3390/biom15091305/s1, Supporting Information included NMR and HRMS spectra of IP1-IP13.
