# Supplementary material for: Isoniazid-Derived Hydrazones Featuring Piperazine/Piperidine Rings: Design, Synthesis, and Investigation of Antitubercular Activity
Source: Biomolecules. 2025 Sep 11;15(9):1305. doi: 10.3390/biom15091305 (PMC12467433; doi:10.3390/biom15091305)

Isoniazid-Derived Hydrazones Featuring Piperazine/Piperidine Rings: Design, Synthesis, and Investigation of Antitubercular Activity

Esma Özcan [1], Siva Krishna Vagolu [2,3*], Rasoul Tamhaev [4,5], Christian Lherbet [4], Lionel Mourey [5], Tone Tønjum [2,6], Miyase Gözde Gündüz [7] and Şengül Dilem Doğan [1*]

[1] Department of Basic Sciences, Faculty of Pharmacy, Erciyes University, 38039 Kayseri, Turkey

[2] Unit for Genome Dynamics, Department of Microbiology, University of Oslo, N-0316, Oslo, Norway

[3] Research Institute of Internal Medicine, Oslo University Hospital, 0027 Oslo, Norway

[4] Laboratoire de Synthèse et Physico-Chimie de Molécules d'Interêt Biologique, LSPCMIB, Université de Toulouse, CNRS, 118 Route de Narbonne, 31062 Toulouse, France

[5] Institut de Pharmacologie et de Biologie Structurale, Université de Toulouse, CNRS, 205 route de Narbonne, BP 64182, 31077 Toulouse Cedex 4, France

[6] Unit for Genome Dynamics, Department of Microbiology, Oslo University Hospital, N-0424, Oslo, Norway

[7] Department of Pharmaceutical Chemistry, Faculty of Pharmacy, Hacettepe University, Sıhhiye, 06100, Ankara, Turkey

*Corresponding authors

Dr. Siva Krishna Vagolu,

Unit for Genome Dynamics, Department of Microbiology, University of Oslo,

N-0316, Oslo, Norway; E-mail address: s.k.vagolu@medisin.uio.no

Dr. Şengül Dilem Doğan,

Erciyes University, Faculty of Pharmacy, Department of Basic Sciences, 38039 Kayseri, TURKEY; E-mail address: dogandilem@gmail.com;

Supporting Information includes ^{1}H NMR, ^{13}C NMR, and HRMS spectra of compounds **IP1–IP13**.

^{1}H NMR, ^{13}C NMR and HRMS spectra of **IP1**

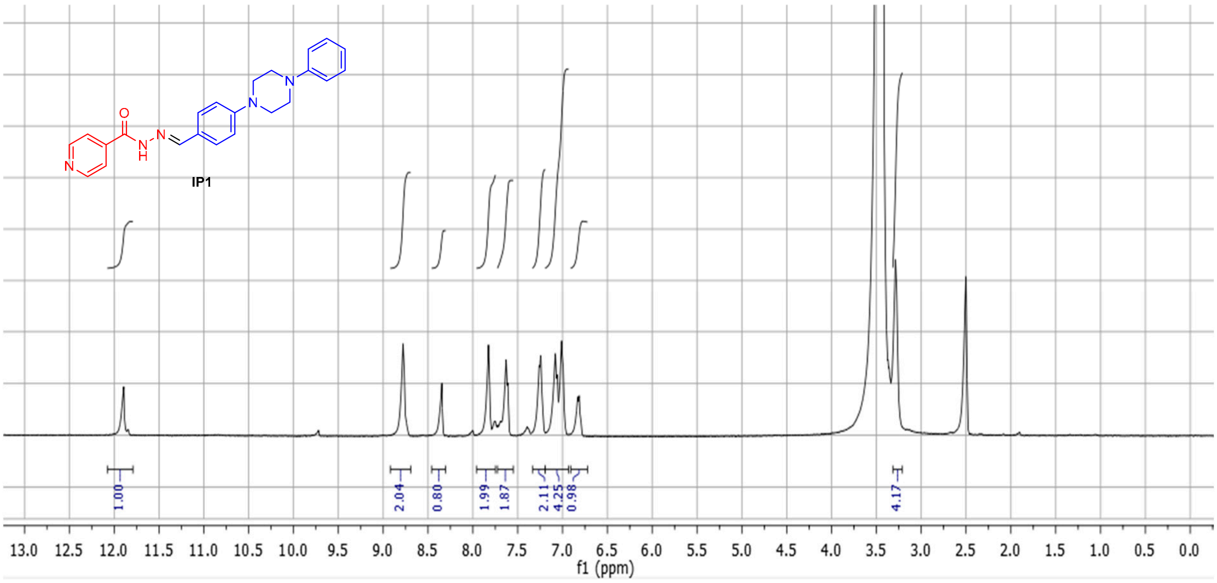

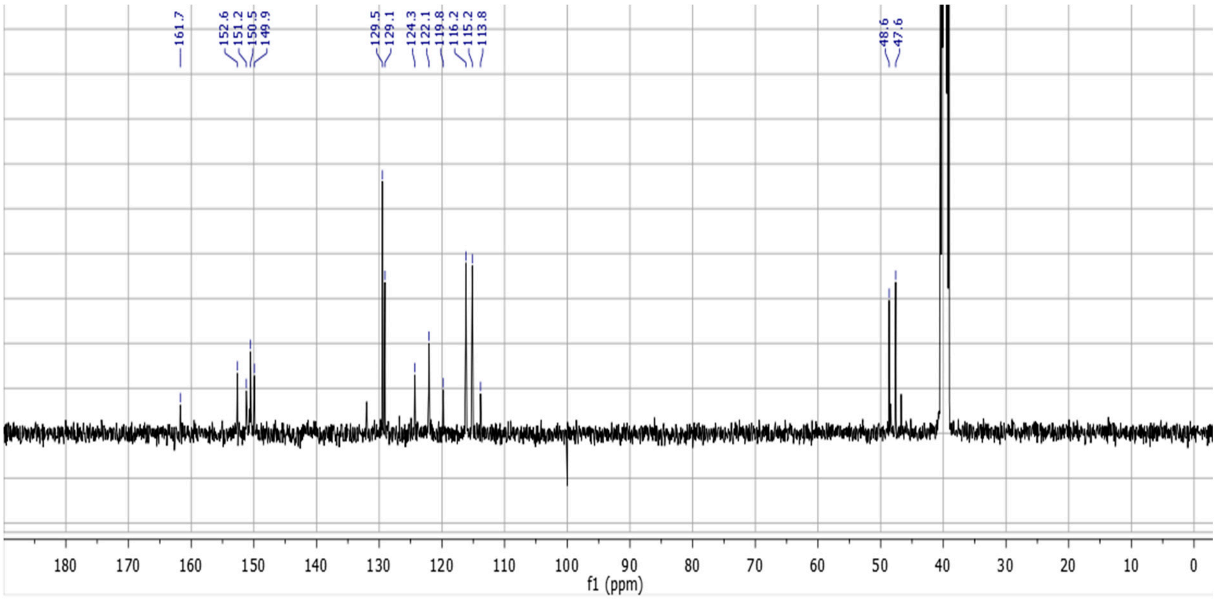

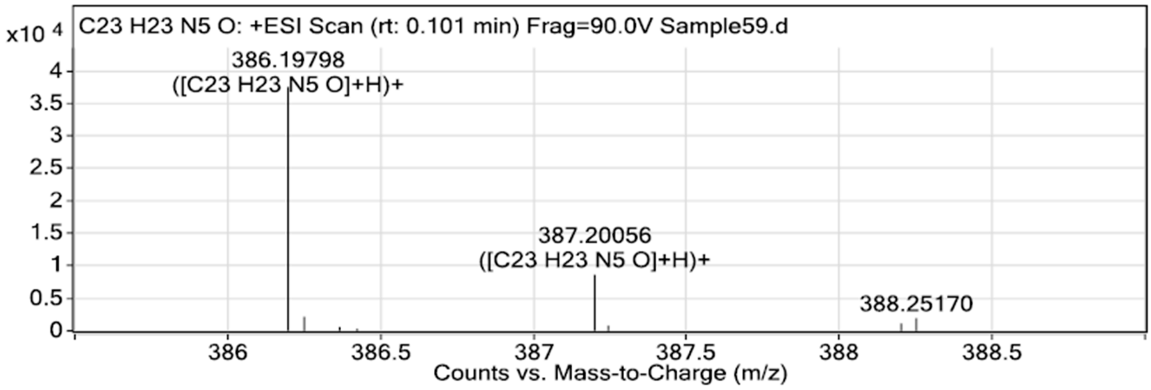

^{1}H NMR, ^{13}C NMR and HRMS spectra of **IP2**

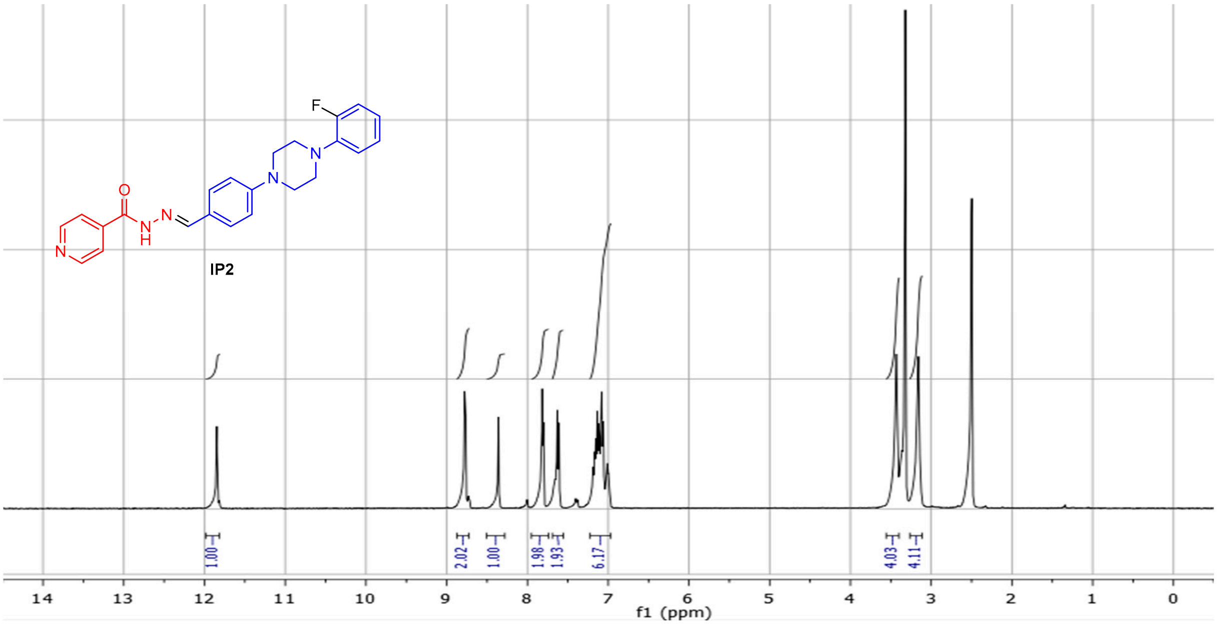

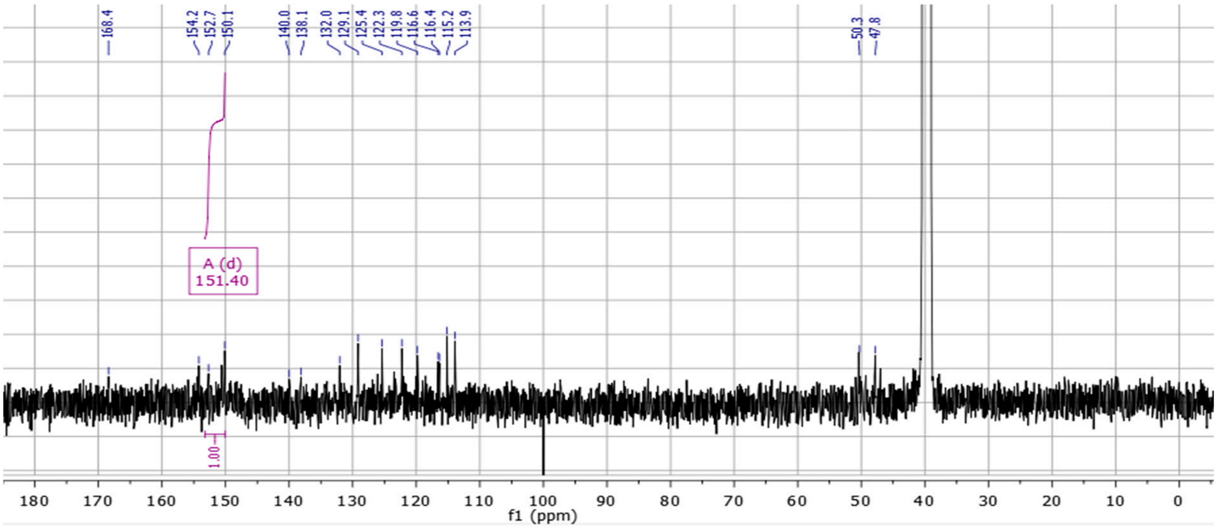

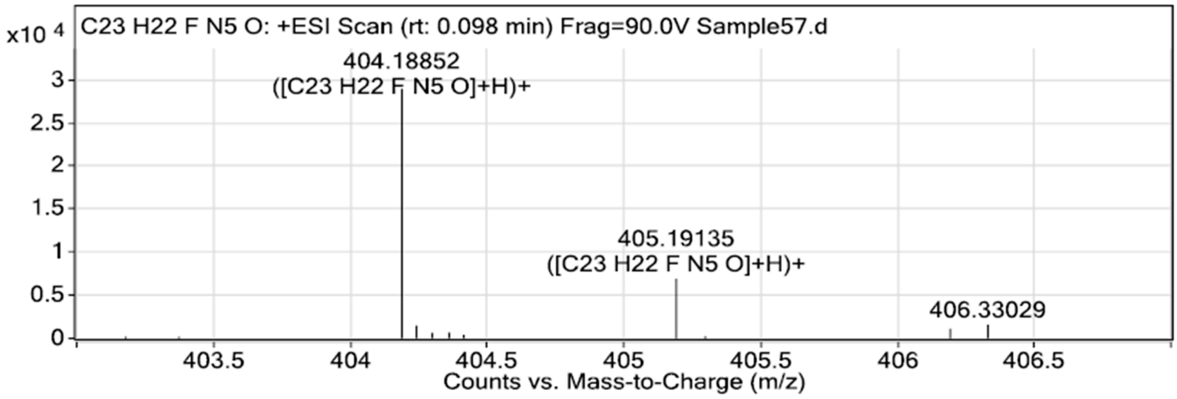

^{1}H NMR, ^{13}C NMR and HRMS spectra of **IP3**

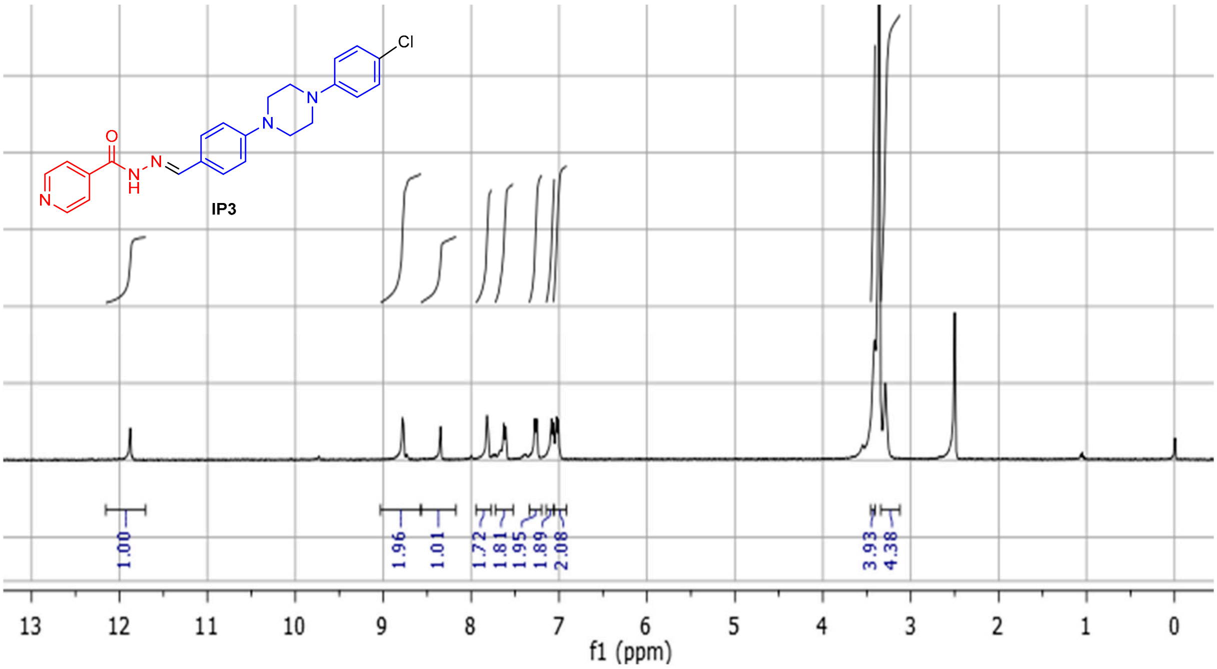

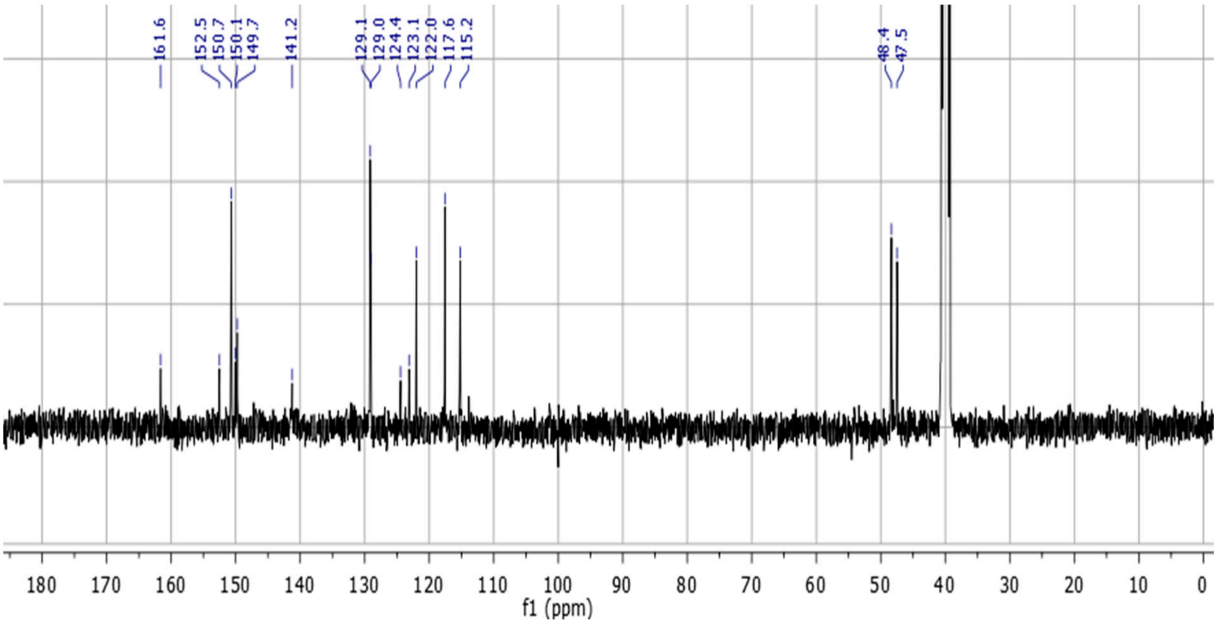

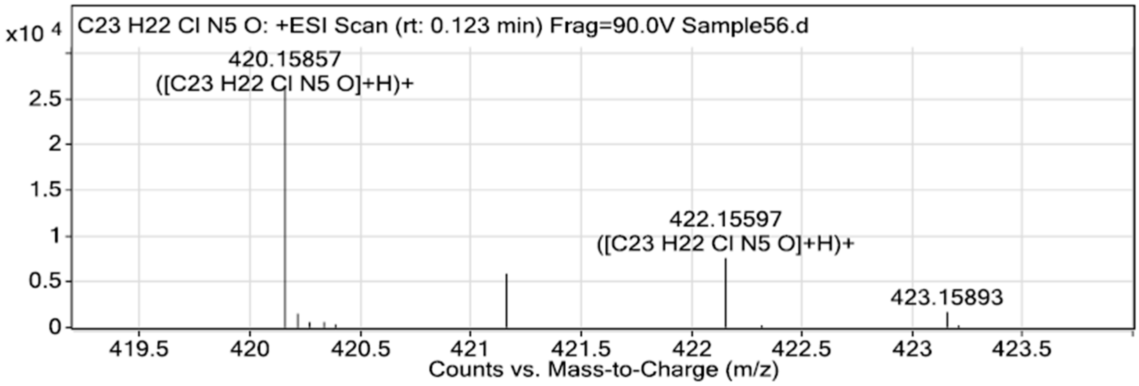

^{1}H NMR, ^{13}C NMR and HRMS spectra of **IP4**

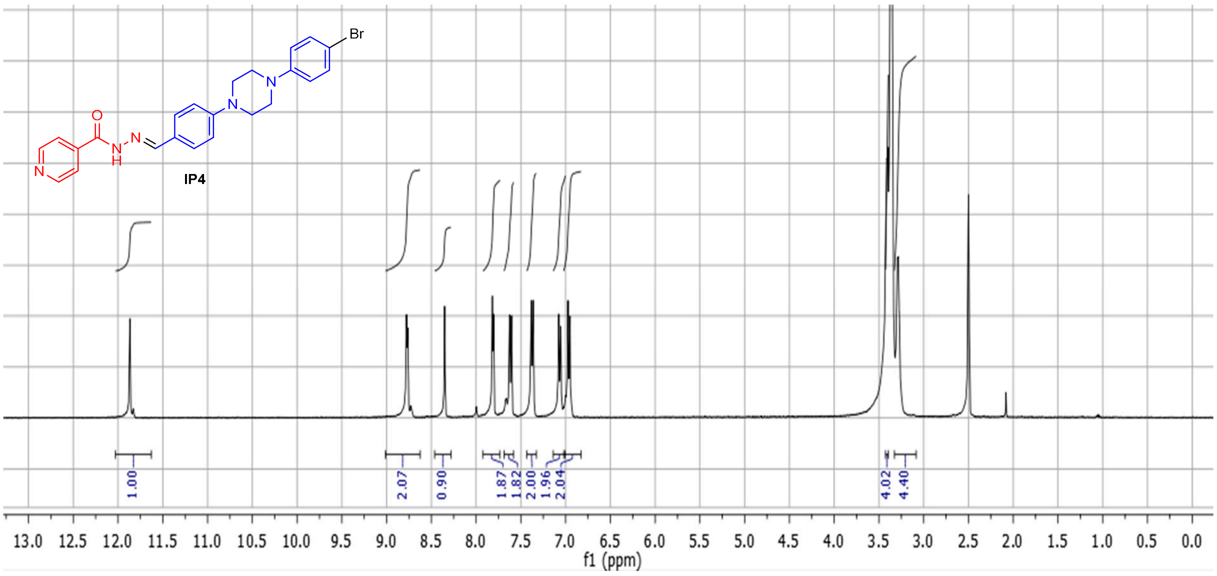

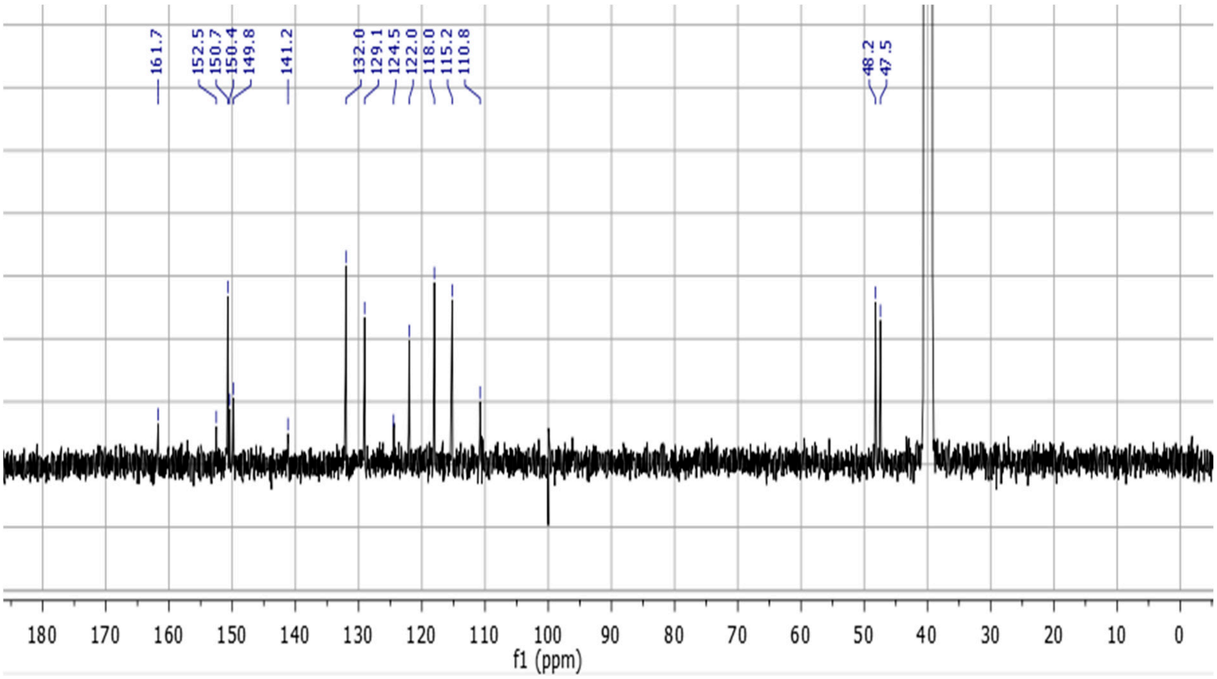

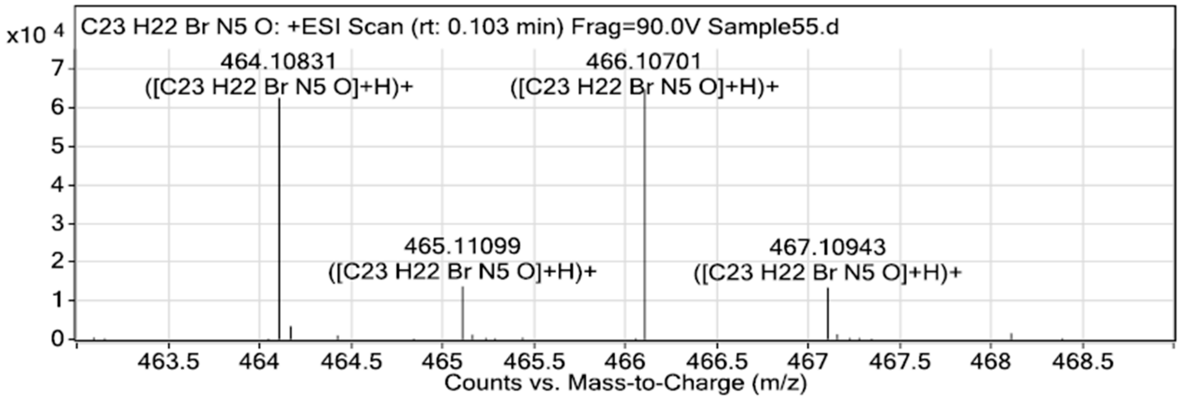

^{1}H NMR, ^{13}C NMR and HRMS spectra of **IP5**

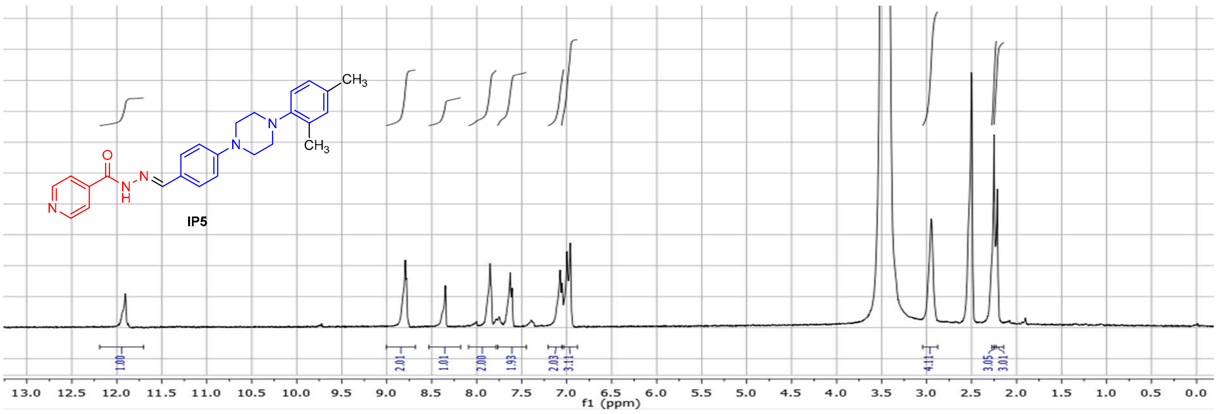

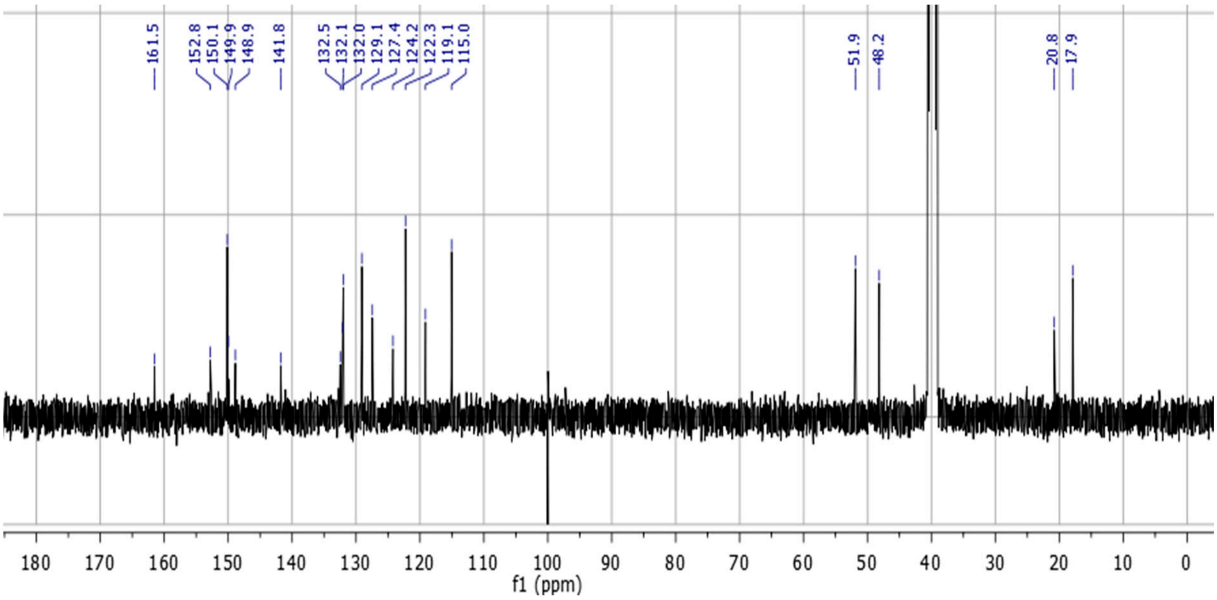

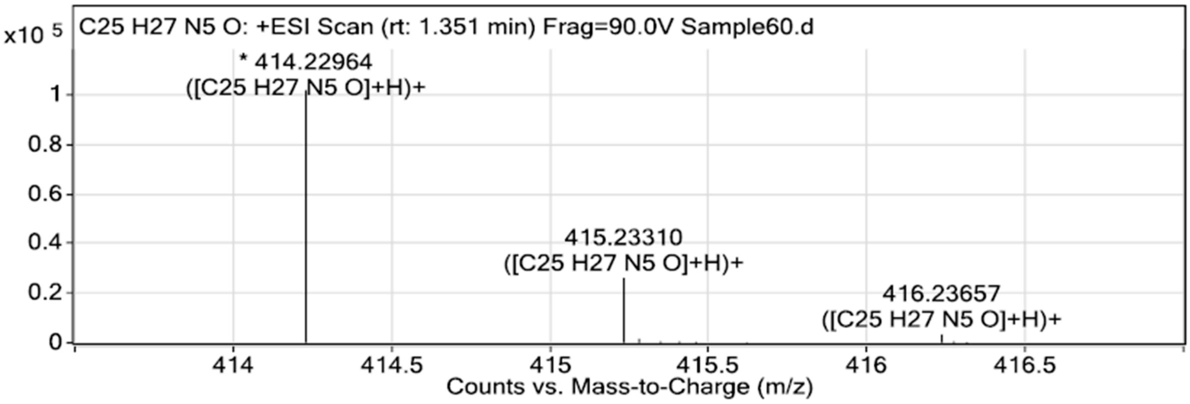

^{1}H NMR, ^{13}C NMR and HRMS spectra of **IP6**

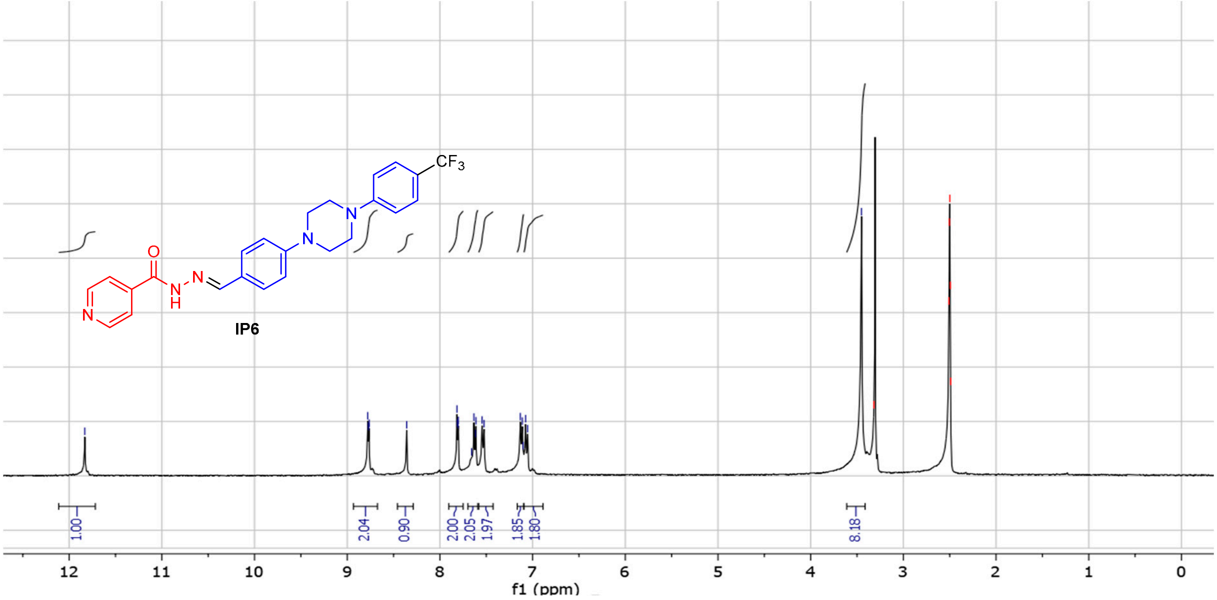

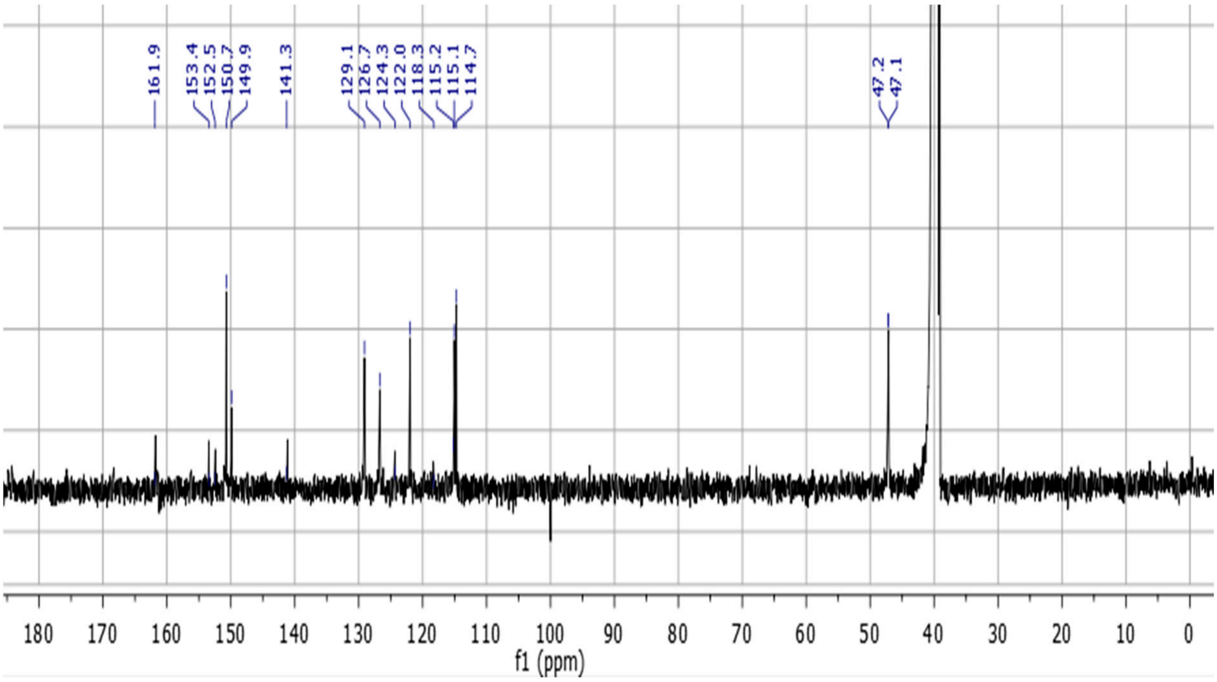

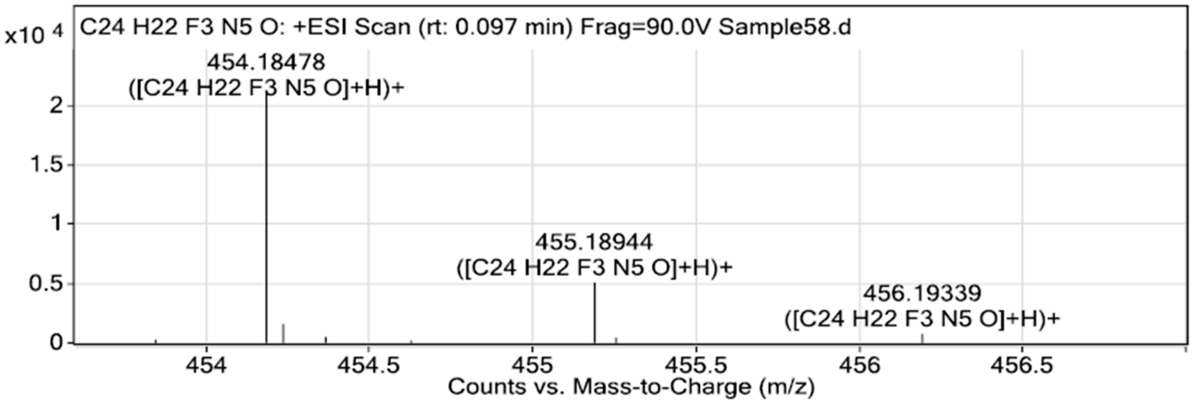

^{1}H NMR, ^{13}C NMR and HRMS spectra of **IP7**

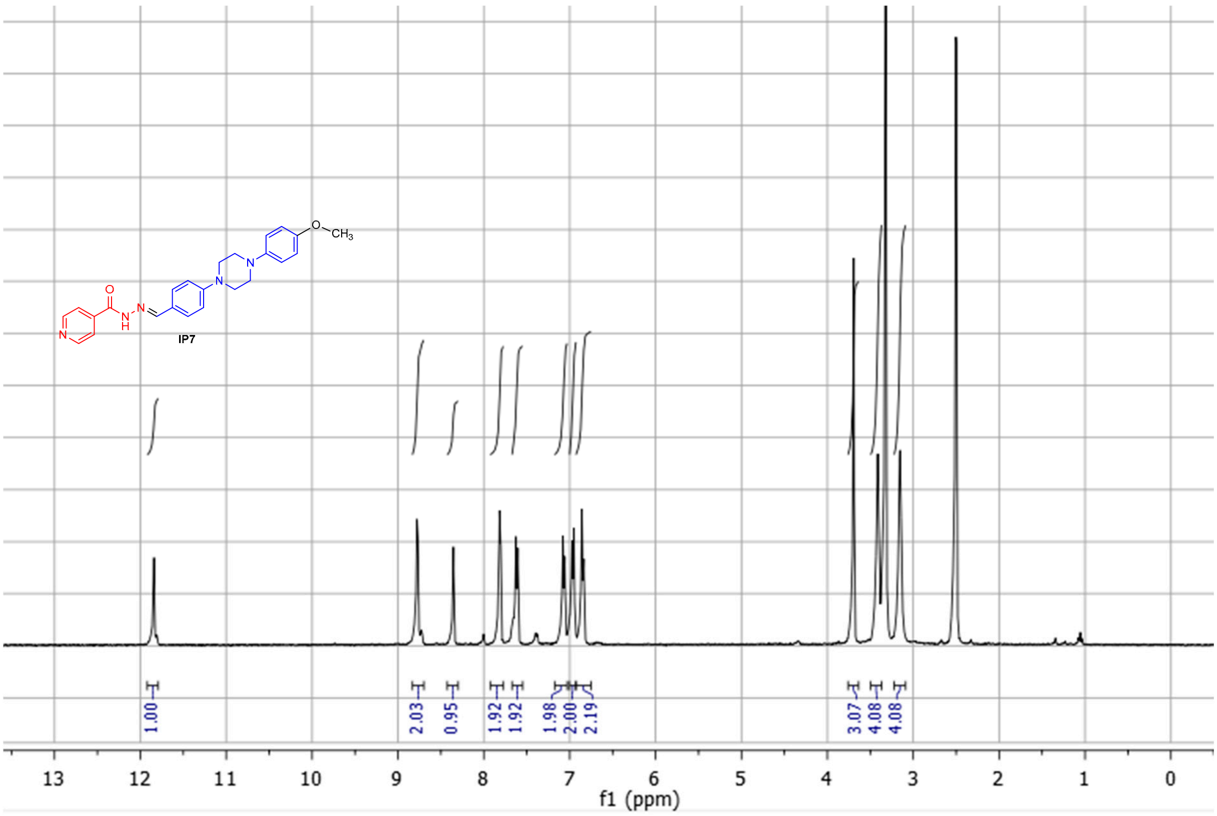

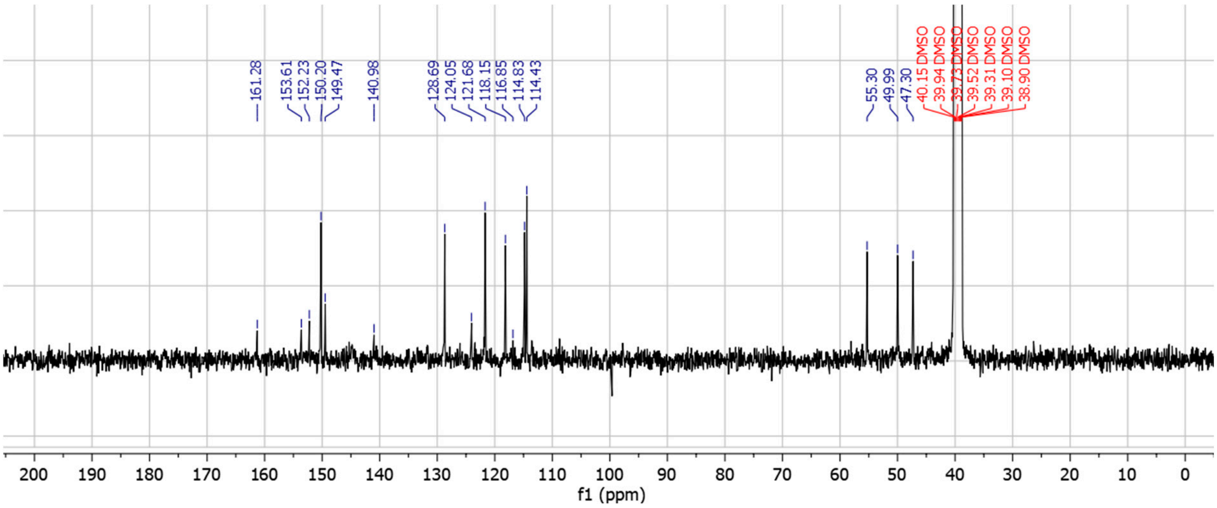

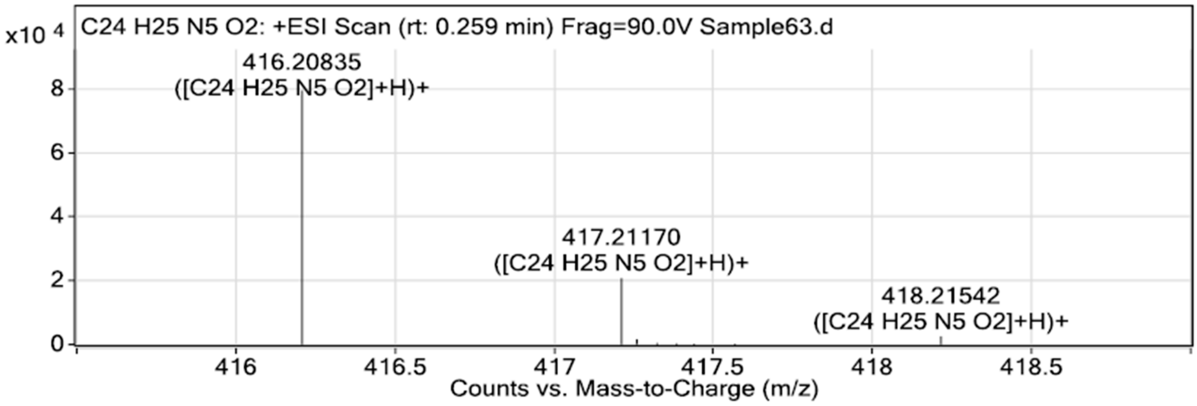

^{1}H NMR, ^{13}C NMR and HRMS spectra of **IP8**

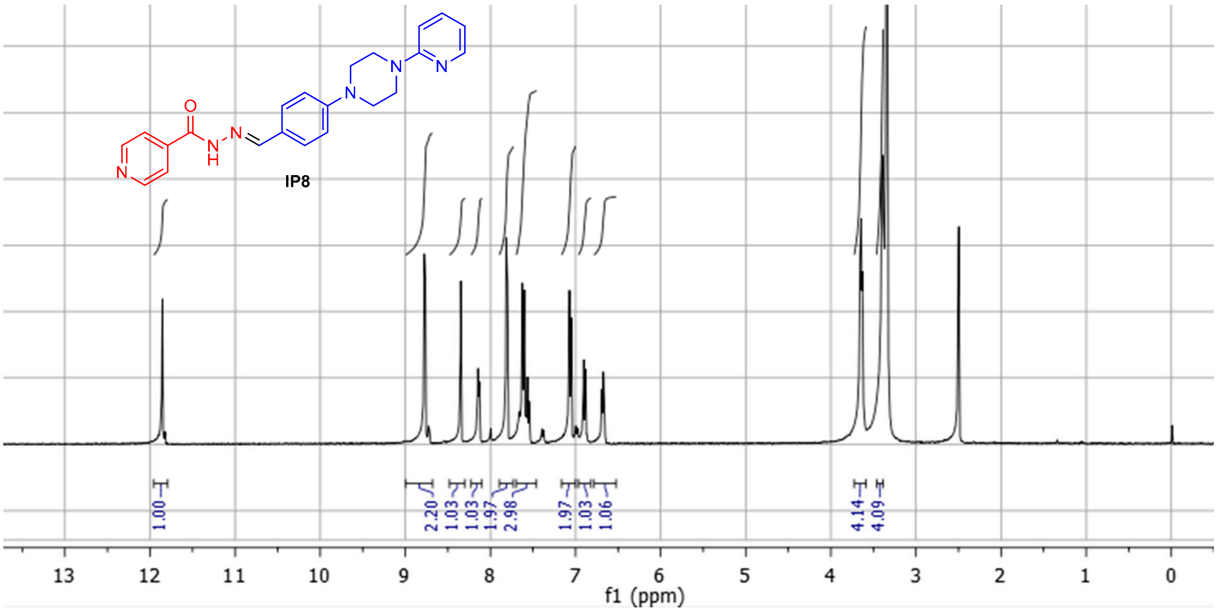

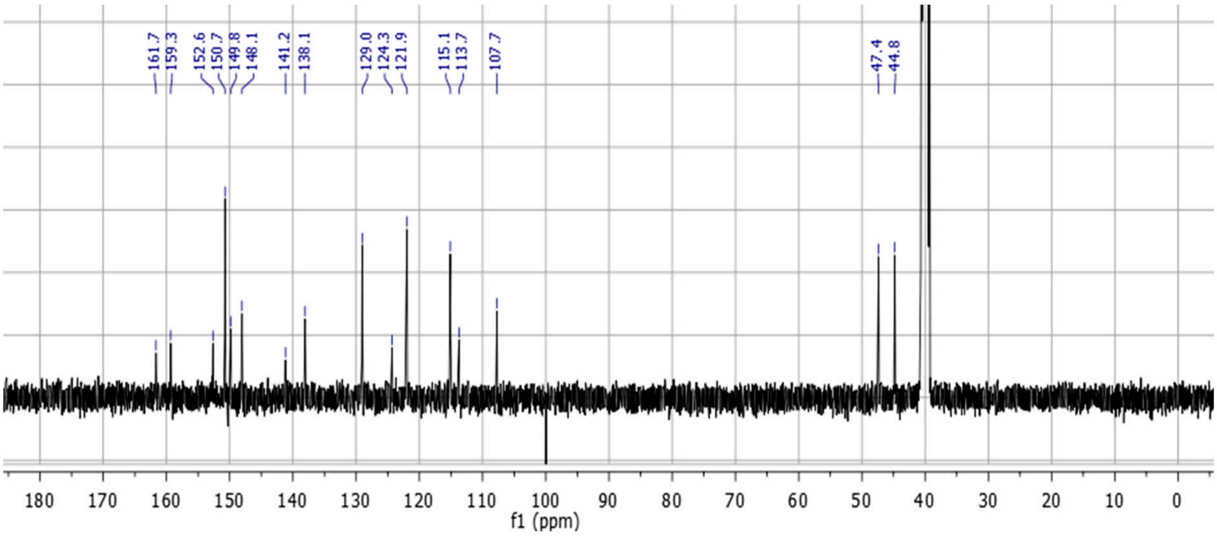

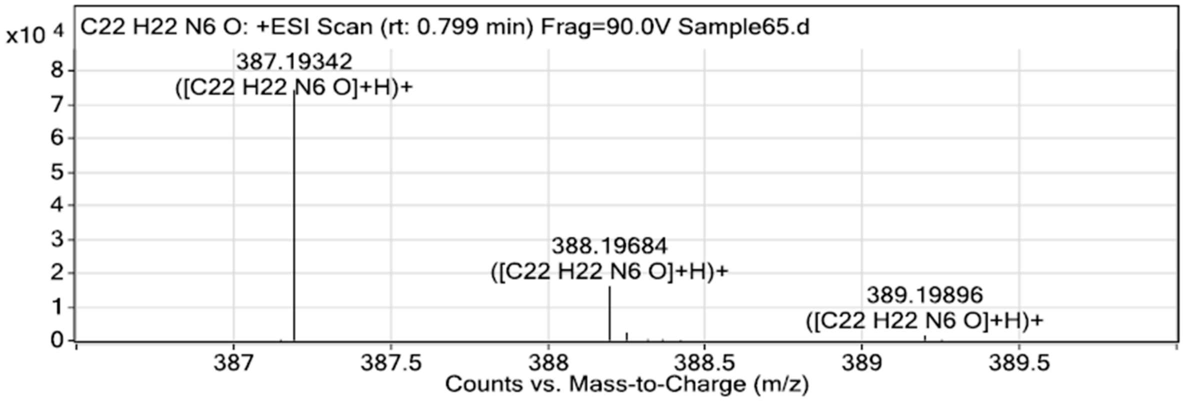

^{1}H NMR, ^{13}C NMR and HRMS spectra of **IP9**

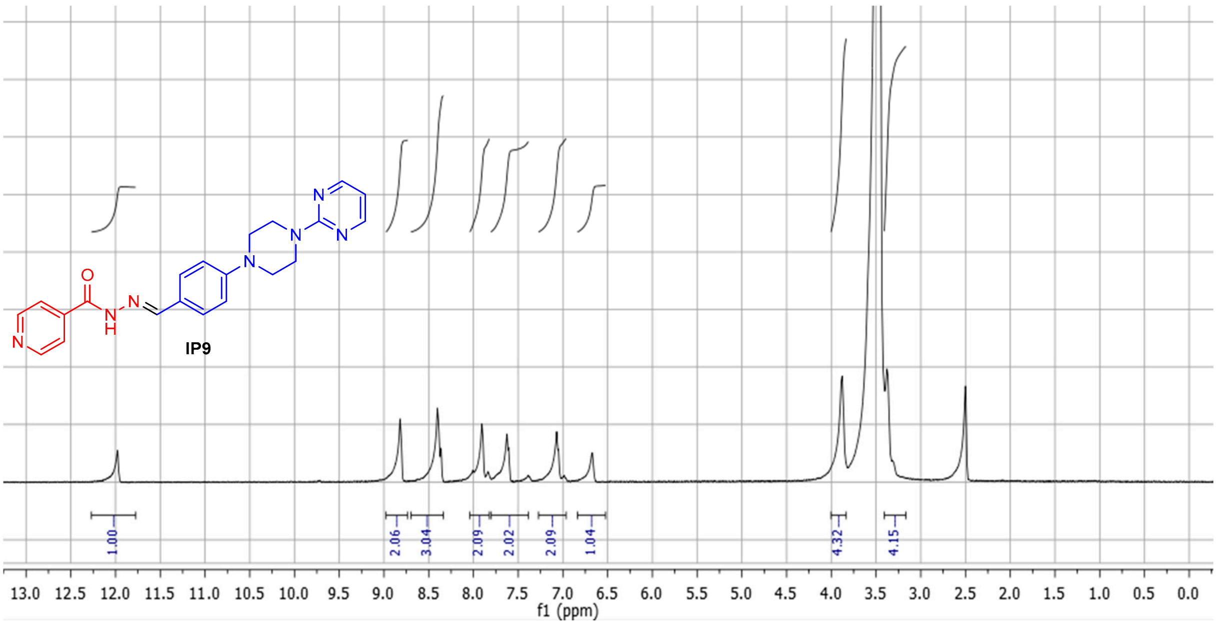

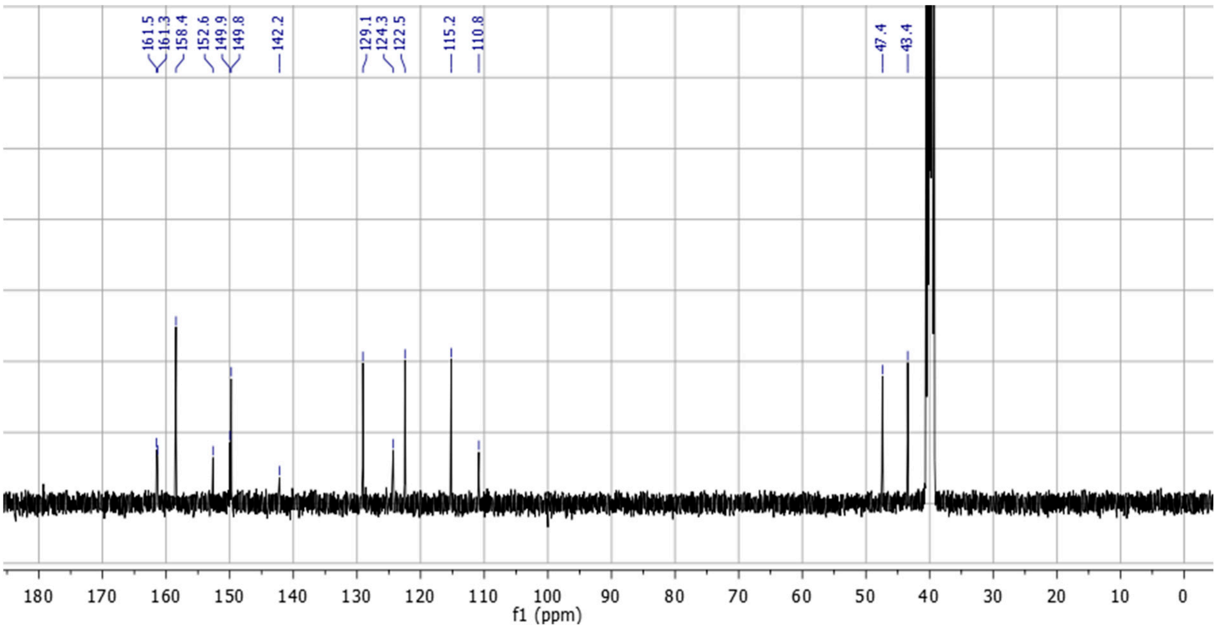

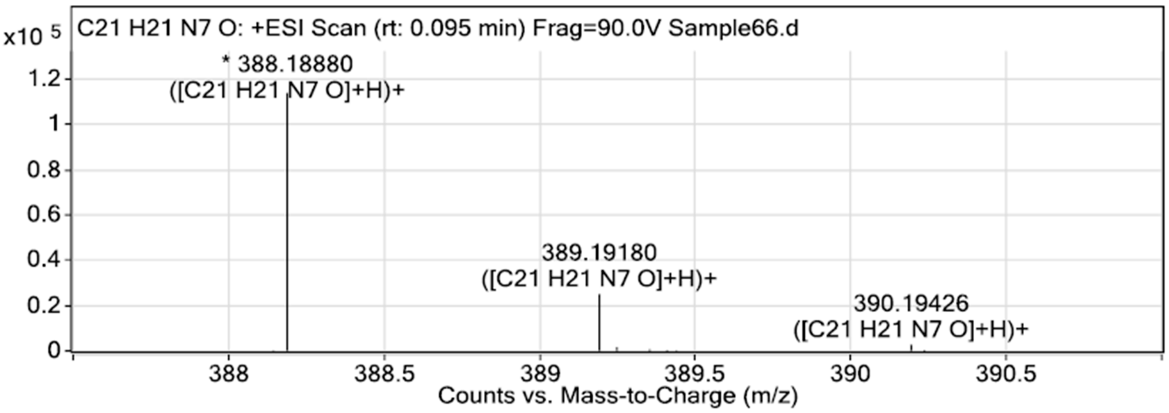

^{1}H NMR, ^{13}C NMR and HRMS spectra of **IP10**

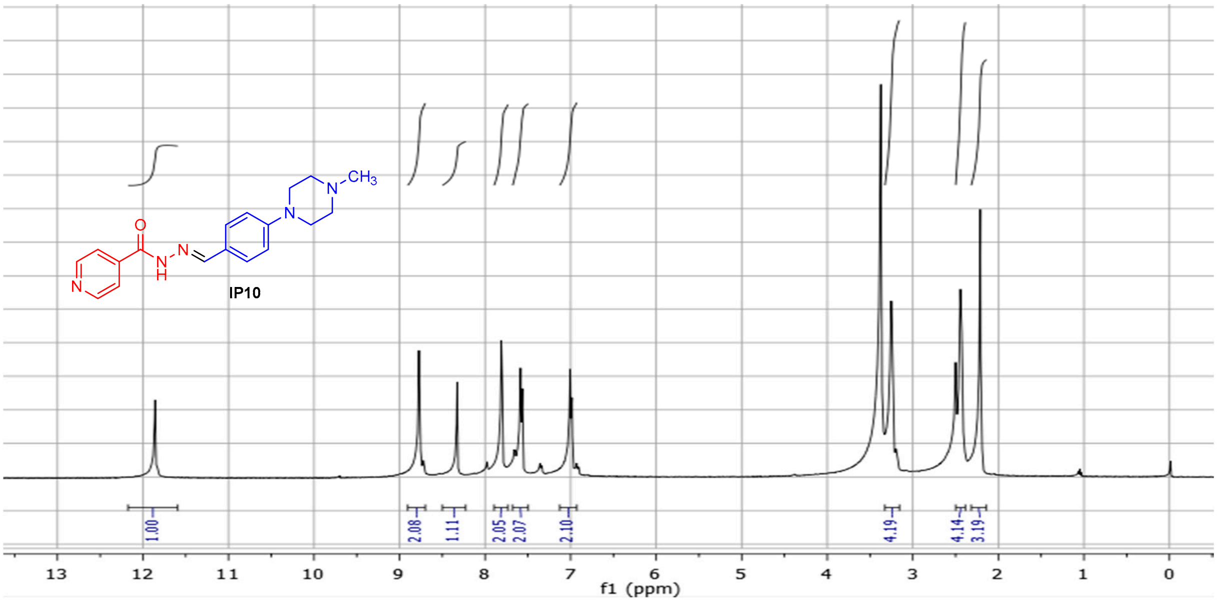

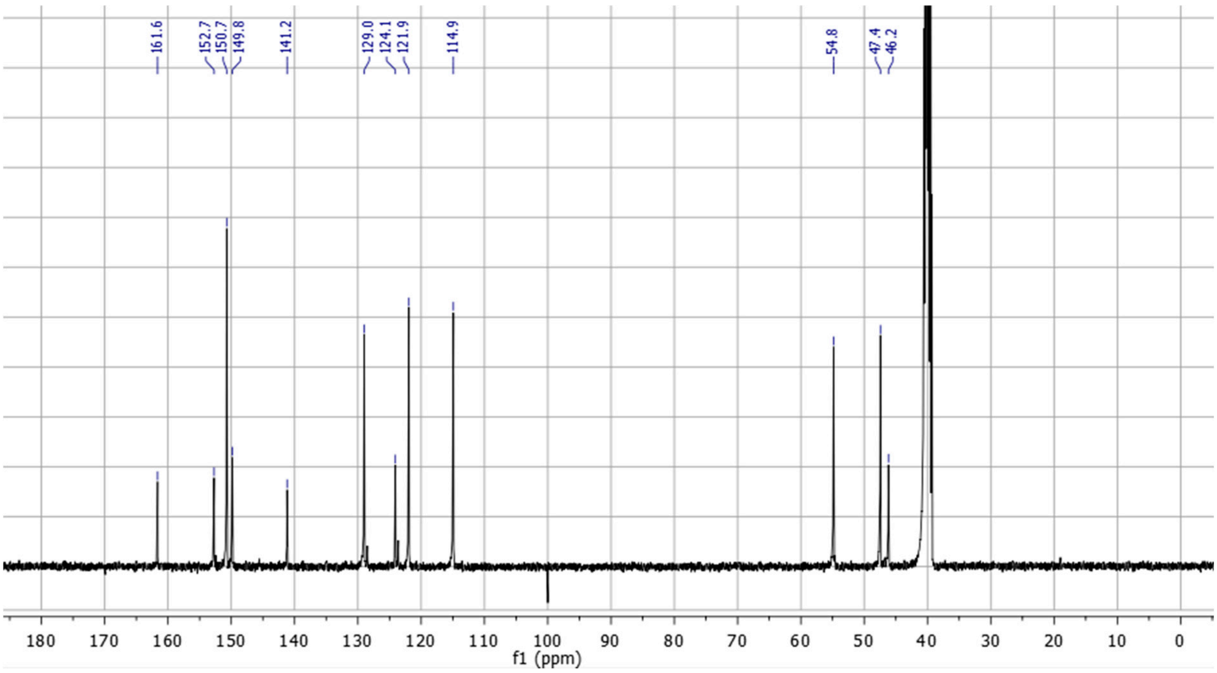

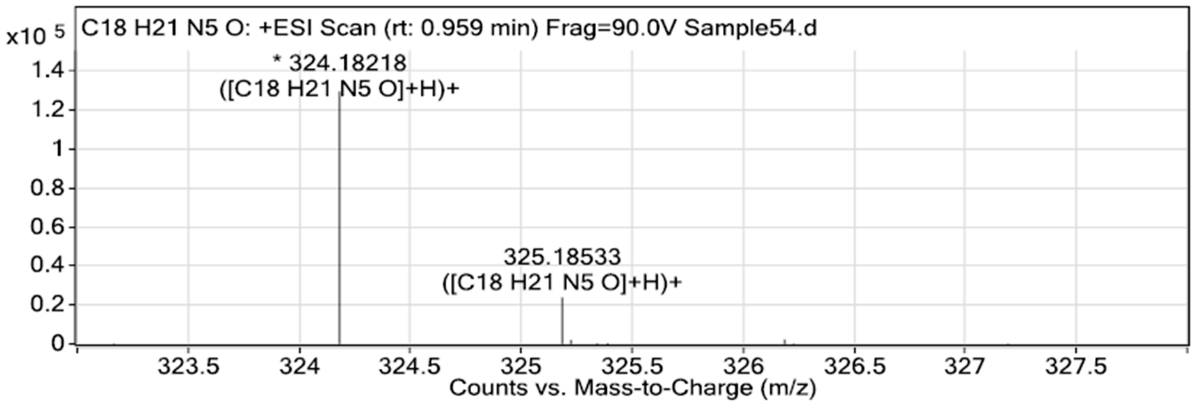

^{1}H NMR, ^{13}C NMR and HRMS spectra of **IP11**

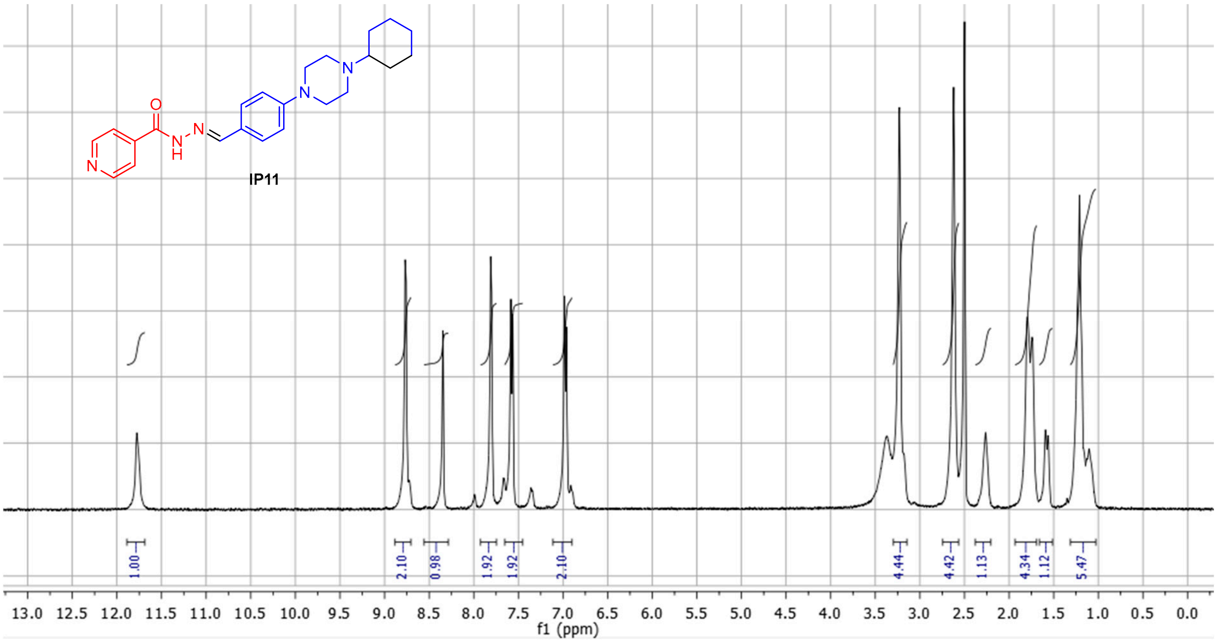

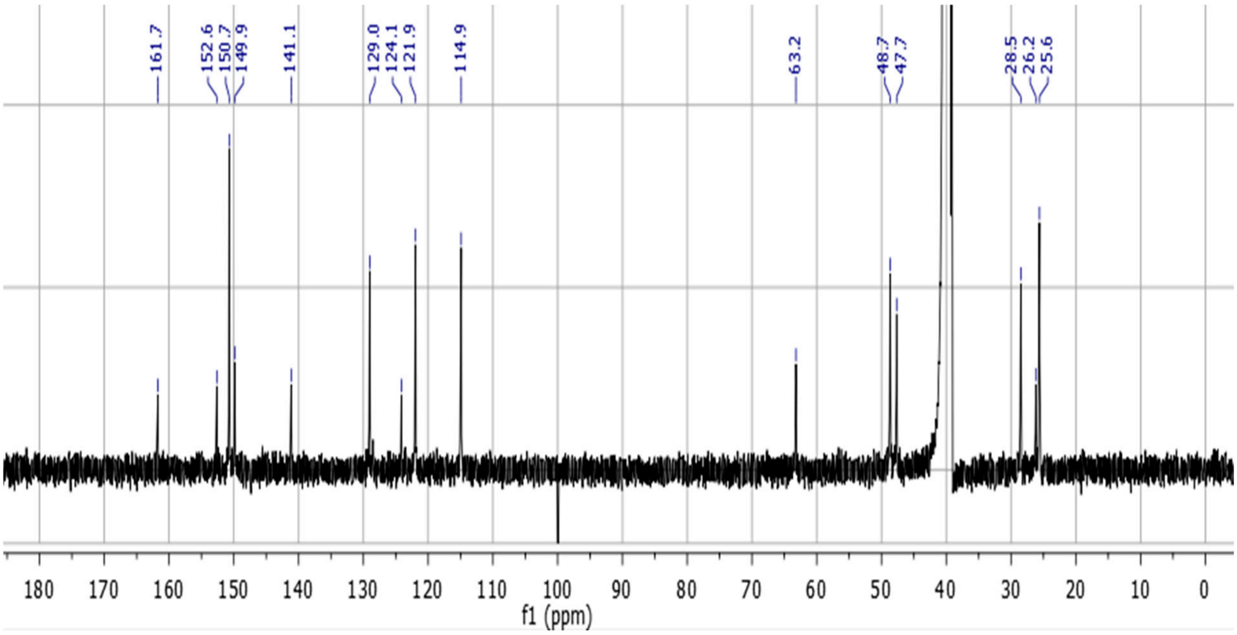

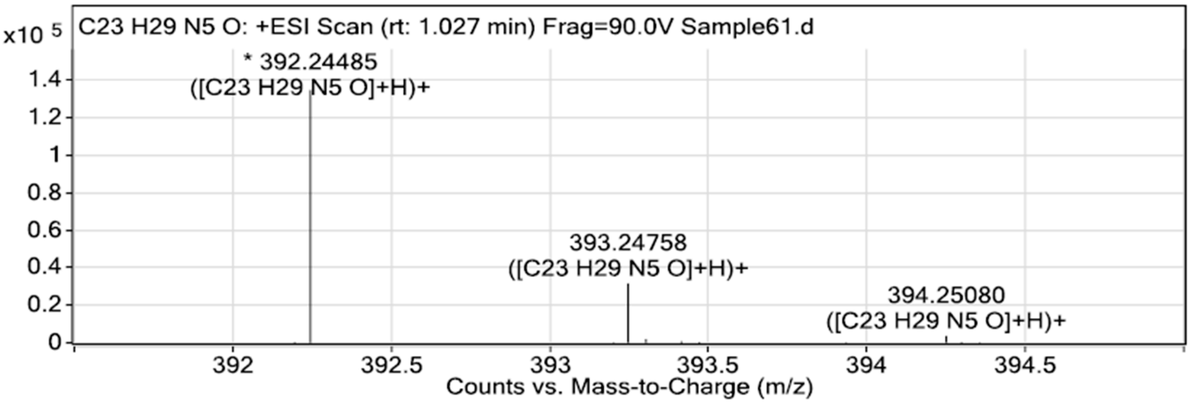

^{1}H NMR, ^{13}C NMR and HRMS spectra of **IP12**

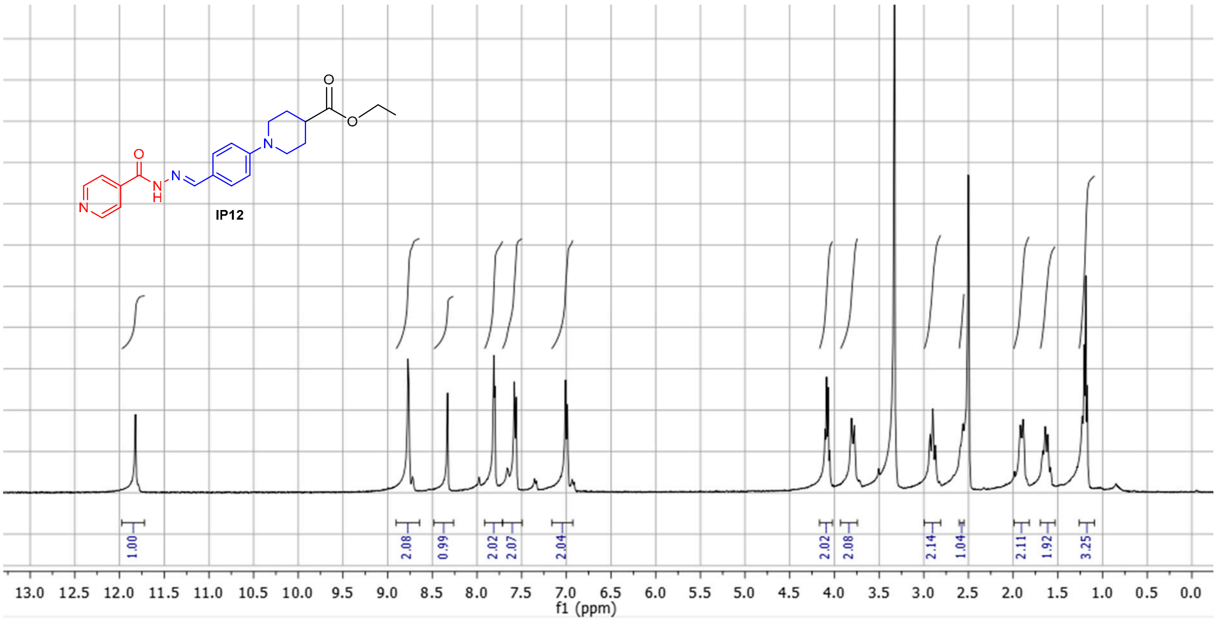

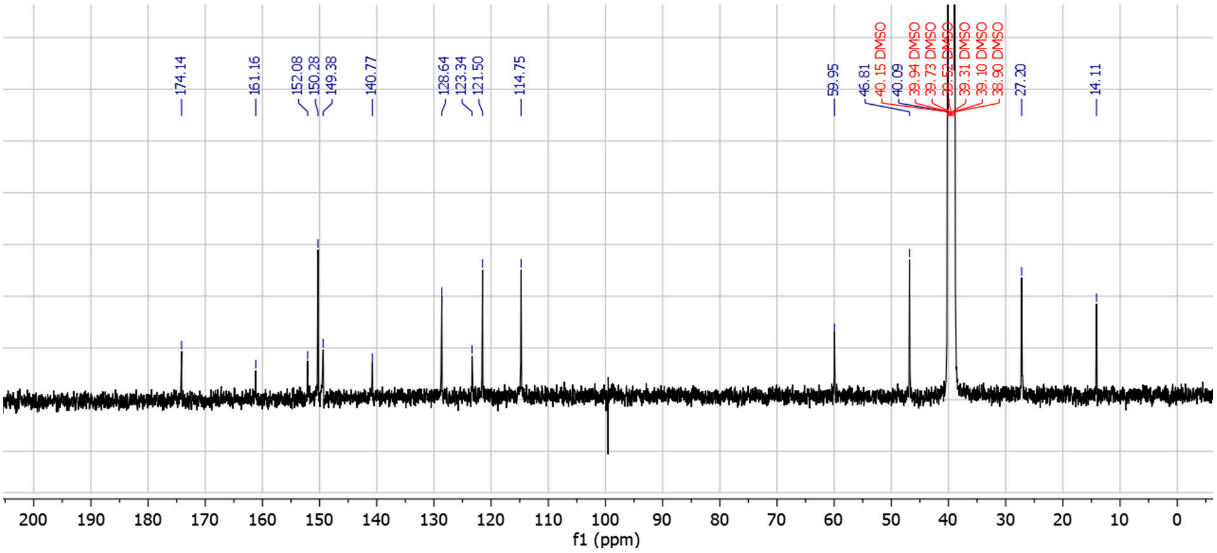

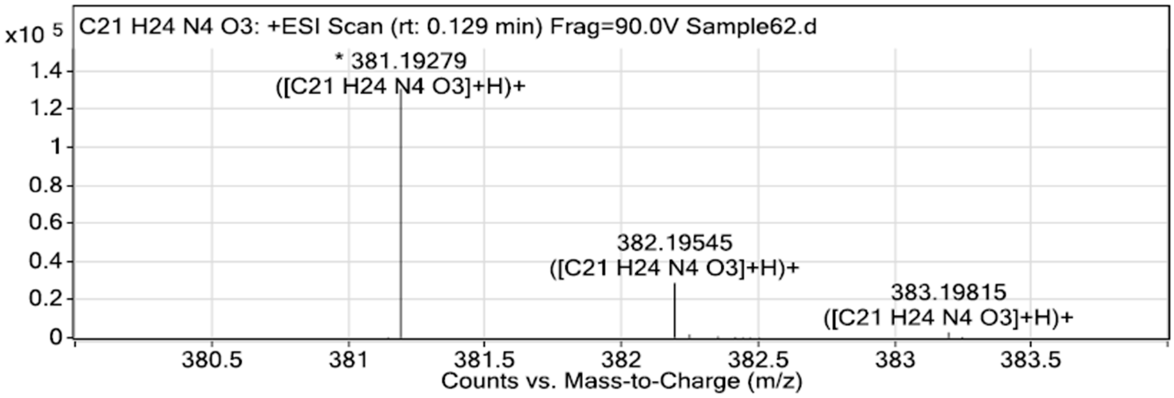

^{1}H NMR, ^{13}C NMR and HRMS spectra of **IP13**

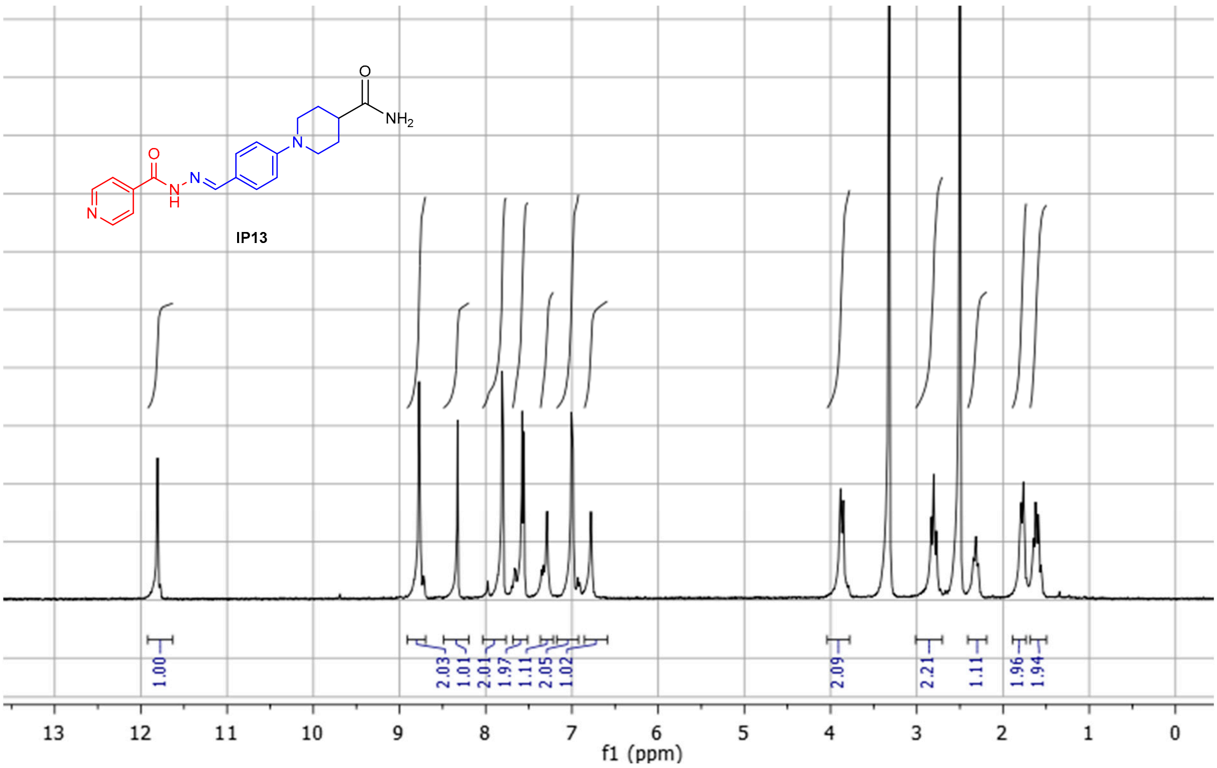

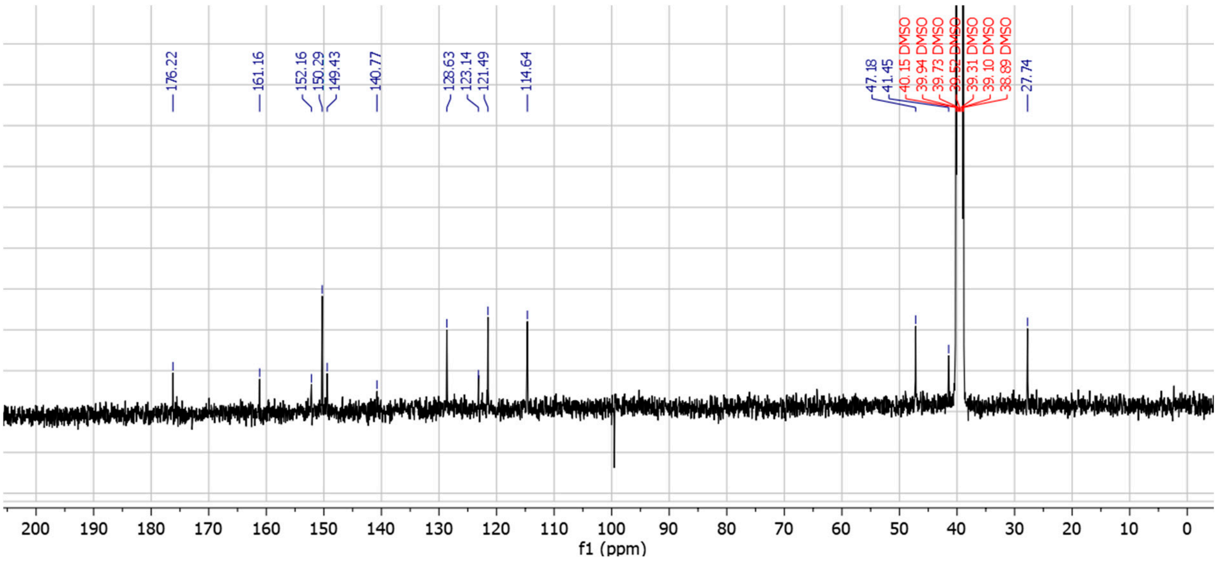

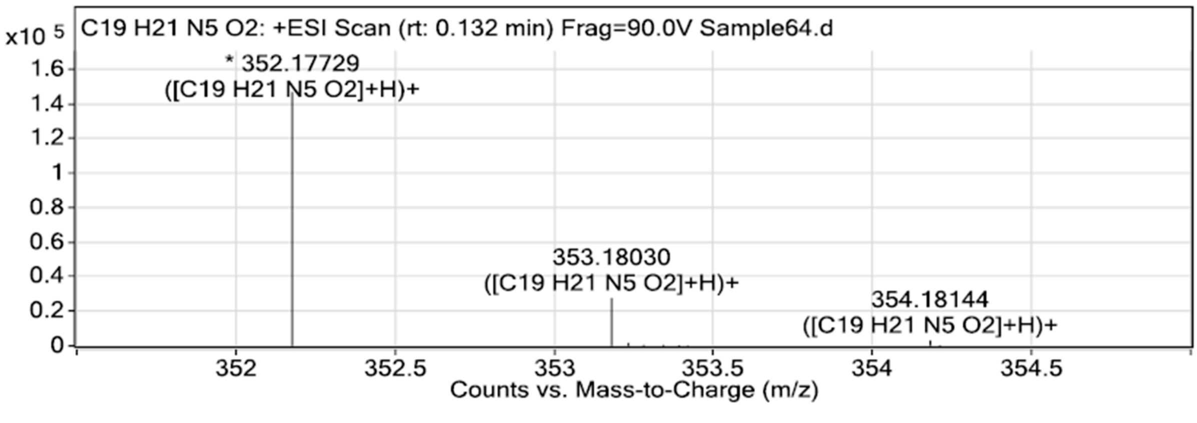

Supplement: Supplementary file 1 [file biomolecules-15-01305-s001.zip › biomolecules-3752076-supplementary.pdf]
